# Bayesian models for aggregate and individual patient data component network meta‐analysis

**DOI:** 10.1002/sim.9372

**Published:** 2022-03-08

**Authors:** Orestis Efthimiou, Michael Seo, Eirini Karyotaki, Pim Cuijpers, Toshi A. Furukawa, Guido Schwarzer, Gerta Rücker, Dimitris Mavridis

**Affiliations:** ^1^ Institute of Social and Preventive Medicine University of Bern Bern Switzerland; ^2^ Department of Psychiatry University of Oxford Oxford UK; ^3^ Institute of Primary Health Care (BIHAM) Université of Bern Bern Switzerland; ^4^ Graduate School for Health Sciences University of Bern Bern Switzerland; ^5^ Department of Global Health and Social Medicine Harvard Medical School Boston Massachusetts USA; ^6^ Department of Clinical Neuro‐ and Developmental Psychology Vrije Universiteit Amsterdam Amsterdam The Netherlands; ^7^ Amsterdam Public Health Research Institute Amsterdam The Netherlands; ^8^ Departments of Health Promotion and Human Behavior and of Clinical Epidemiology Kyoto University Graduate School of Medicine/School of Public Health Kyoto Japan; ^9^ Institute of Medical Biometry and Statistics, Faculty of Medicine and Medical Center University of Freiburg Freiburg Germany; ^10^ Department of Primary Education University of Ioannina Ioannina Greece; ^11^ Faculté de Médecine Université Paris Descartes Paris France

**Keywords:** complex interventions, composite, model selection, multiple treatments

## Abstract

Network meta‐analysis can synthesize evidence from studies comparing multiple treatments for the same disease. Sometimes the treatments of a network are complex interventions, comprising several independent components in different combinations. A component network meta‐analysis (CNMA) can be used to analyze such data and can in principle disentangle the individual effect of each component. However, components may interact with each other, either synergistically or antagonistically. Deciding which interactions, if any, to include in a CNMA model may be difficult, especially for large networks with many components. In this article, we present two Bayesian CNMA models that can be used to identify prominent interactions between components. Our models utilize Bayesian variable selection methods, namely the stochastic search variable selection and the Bayesian LASSO, and can benefit from the inclusion of prior information about important interactions. Moreover, we extend these models to combine data from studies providing aggregate information and studies providing individual patient data (IPD). We illustrate our models in practice using three real datasets, from studies in panic disorder, depression, and multiple myeloma. Finally, we describe methods for developing web‐applications that can utilize results from an IPD‐CNMA, to allow for personalized estimates of relative treatment effects given a patient's characteristics.

## 1 INTRODUCTION

Network meta‐analysis (NMA) is a statistical technique that generalizes the usual (pairwise) meta‐analysis. NMA can jointly synthesize evidence from multiple studies comparing different sets of treatments for the same disease.^1^, ^2^, ^3^ In standard applications of NMA, each node of the network corresponds to a single, independent treatment. Sometimes, however, the nodes of a network are complex interventions (ie, combinations of a set of basic components). For example, a network on psychological treatments for panic disorder included studies that examined cognitive behavioral therapy (CBT)^4^; CBT was however administered in many different formats, for example, with or without a muscle relaxation component, with or without breathing retaining and so forth. These components were also included in other treatments, for example, physiological therapy. In such cases, it is reasonable to expect that the effectiveness of a treatment will depend on the exact components that it included, and it is of high clinical relevance to identify the most efficient components.

Such data can be analyzed using a component network meta‐analysis (CNMA) model. This model was first proposed by Welton et al,^5^ and is an extension of NMA that takes into account the way different components are combined to make up the interventions of the network. The model by Welton et al was developed in a Bayesian setting, and Rücker et al^6^ implemented it in a frequentist setting. The frequentist version of CNMA is included in the **netmeta** ^7^ package in R, via the netcomb function. Α recent paper by Petropoulou et al^8^ provided an overview of the methodology. A CNMA model can be used (in principle) to estimate the individual effect of each component, and to identify the most beneficial components. One attractive feature of this model is that it can be implemented even if the network is disconnected at the treatment level^6^ (ie, it consists of two or more subnetworks with no common nodes) so long as some components are common across subnetworks. Another characteristic, which is unique in evidence synthesis, is that this model can be used to design new treatments. For example, if the model identifies a set of components to be beneficial, the best treatment according to the model will be the combination of these components; this may be a completely new combination, that is, a treatment previously not tested in a trial. This new treatment can then be tested in a new trial, provided of course that we are able to put these components together in practice.

The simplest and most common assumption of a CNMA model is additivity, that is, to assume that each component acts independently of others. In real applications, however, there may be interactions between at least some of the components. Welton et al described^5^ how to extend the CNMA model to account for such interactions, and this is also incorporated in **netmeta**. However, when the number of components is large, there are numerous interactions that can be included in the model and including all possible interactions would lead to large uncertainty in the estimates. Thus, a method for selecting potentially relevant interactions to include in a CNMA model is of interest. Rücker et al^9^ recently described forward and backward stepwise strategies for selecting interactions. The advantages of these methods are that they are relatively objective, they are easy to apply, easy to replicate, and they lead to simple models. On the other hand, the use of stepwise methods for variable selection in regression modeling in general has been widely criticised.^10^, ^11^ Relatively more recent methods for variable selection are based on shrinking regression coefficients, for example, as in LASSO.^12^ There is, however, currently no guidance for applying shrinkage methods for selecting interaction terms in CNMA. Moreover, individual patient data (IPD) NMA is becoming increasingly popular,^13^ while the available CNMA models can only handle aggregate (study‐level) data.

In this article, we aim to address these gaps, by presenting a set of Bayesian models for CNMA. Our aggregated data models can identify the most prominent interactions between components without resorting to stepwise selection methods, while incorporating prior information about possibly important interactions. Our IPD models can additionally include component‐covariate interactions, and their results can be used to obtain patient‐specific estimates of relative treatment effects between any two combinations of components. We illustrate all methods in three real clinical examples, and we discuss how to utilize results from IPD CNMA models to develop web‐applications that can provide patient‐level estimates of relative treatment effects.

## 2 EXAMPLE DATASETS

We present here two real datasets to illustrate our methods. In the Appendix, we describe one additional example from multiple myeloma.

### 2.1 Psychotherapies for panic disorder (aggregate data only)

This dataset comprises study‐level information from 60 RCTs in patients suffering from panic disorder. The interventions were psychological therapies, and active treatments were compared with each other or with control interventions. The outcome we focus on is short‐term binary remission of panic disorder at 3 months. The treatments were combinations of 12 components: wl (waiting list), pl (placebo), ftf (face‐to‐face), pe (psychoeducation), ps (psychological support), br (breathing retaining), mr (progressive/applied muscle relaxation), ive (in vivo exposure), ine (interoceptive exposure), vre (virtual reality exposure), cr (cognitive restructuring), and w3 (third wave). This dataset has been described in more detail elsewhere.^4^ We show the network in the Appendix, and we provide the data in GitHub.

### 2.2 Internet delivered psychological therapies for depression (aggregate and individual patient data)

This dataset includes data from RCTs on people with depression, comparing several internet‐based psychotherapies with each other or with inactive controls. The outcome of interest is depression symptom severity, measured on the Patient Health Questionnaire‐9 (PHQ‐9). This scale takes values from 0 to 27, with larger values indicating more severe symptoms. For a total of 21 RCTs there were only aggregate (study‐level) data available, that is, mean, SD and number of patients per treatment arm. There were also 49 RCTs that provided patient‐level information on 10 331 patients. In addition to the primary outcome, these 49 studies provided information about baseline severity in PHQ‐9 and gender; all studies except one provided information about age; all studies except one provided information about relationship status (in relationship: yes/no). The treatments were a combination of a set of 17 components: wl (waiting list), dt (drug therapy), pl (treatment effects), pe (psychoeducation), cr (cognitive restructuring), ba (behavioral activation), is (interpersonal skill training), ps (problem solving), re (relaxation), w3 (third wave), bi (behavioral therapy for insomnia), rp (relapse prevention), hw (homework required), ftf (face to face), ae (automated encouragement), he (human encouragement), and tg (therapeutic guidance). More details on the components have been provided elsewhere.^14^, ^15^ The network is shown in the Appendix. Due to restrictions in data sharing agreements, the data cannot be made publicly available.

## 3 METHODS

Here we describe the standard NMA model (ie, at the “treatment level”) and then discuss various modeling approaches for performing the analysis at the “component level.”

### 3.1 Network meta‐analysis at the treatment level

Assume that study i compares treatment X and Y and it provides aggregate data (AD), that is, an estimated treatment effects on a continuous scale (eg, mean difference, standardized mean difference, but also log‐odds/risk ratio, etc.) denoted as $y_{i}$, and the corresponding sampling variance is $s_{i}^{2}$. Then the usual random effects Bayesian NMA model can be written as follows:

**Model I: Aggregate data network meta‐analysis (AD‐NMA)**

$$y_{i}∼N\left(\delta_{\mathrm{YX},i},s_{i}^{2}\right)$$

$$\delta_{\mathrm{YX},i}∼N\left(d_{\mathrm{YA}}-d_{\mathrm{XA}},\tau^{2}\right)$$

$$\tau^{2},d_{\mathrm{XA}},d_{\mathrm{YA}},\ldots ∼\left(\text{prior distributions}\right)$$

where Α is the reference treatment in the network (which can be chosen arbitrarily), and $\tau^{2}$ is the variance of the random effects, assumed common for all pairwise treatment comparisons in the network. The model can be easily modified for the case when arm‐level information is available (ie, mean outcome and SD). Also for binary outcomes, the model can be modified to include a binomial distribution when numbers of events per treatment arm are available. For multi‐arm trials the model needs to be adjusted by including multivariate normal distributions. For more details on the standard AD‐NMA model and its extensions we refer to a comprehensive review.^3^

The NMA model can be readily extended to studies with IPD.^13^, ^16^, ^17^ Assume that for patient k randomized in study i to receive treatment $t_{\mathrm{ik}}$ we observe the outcome $y_{\mathrm{ik}}$. Also assume that for this patient we have information on several covariates included in vector $x_{\mathrm{ik}}\;$(which without loss of generality will be assumed to be centralized using the overall mean of each covariate across the whole dataset). Finally, assume that this study was a two‐arm study comparing treatments X and Y, and that A is the reference. The model is as follows:

**Model II: Individual participant data network meta‐analysis (IPD‐NMA)**

$$y_{\mathrm{ik}}∼N\left(m_{\mathrm{ik}},\sigma_{i}^{2}\right)$$

$$m_{\mathrm{ik}}=\left\{\begin{array}{c} \alpha_{i}+\beta^{\prime }x_{\mathrm{ik}}\;\;\text{if}\;t_{\mathrm{ik}}=X\\ \alpha_{i}+\beta^{\prime }x_{\mathrm{ik}}+{\left(\gamma_{\mathrm{YA}}-\gamma_{\mathrm{XA}}\right)}^{\prime }x_{\mathrm{ik}}+\delta_{\mathrm{YX},i}\;\text{if}\;t_{\mathrm{ik}}=Y \end{array}\right.$$

$$\delta_{\mathrm{YX},i}∼N\left(d_{\mathrm{YA}}-d_{\mathrm{XA}},\tau^{2}\right)$$

$$\gamma_{\mathrm{AA}}=d_{\mathrm{AA}}=0$$

$$\sigma^{2},\tau^{2},\alpha_{i},\beta ,\gamma_{\mathrm{XA}},\gamma_{\mathrm{ZΑ}},\ldots ,d_{\mathrm{XA}},d_{\mathrm{YA}},\ldots ∼\left(\text{prior distributions}\right)$$

In this expression, β′ is the transpose of β, the vector of coefficients that encapsulate the prognostic power of x. Vector $\gamma_{\mathrm{XA}}$ includes the regression coefficients for effect modifications (treatment‐covariate interactions), for treatment X vs A. Parameter $d_{\mathrm{XA}}$ estimates the relative treatment effects for X vs A at x=0. Unexplained variability in the outcome is included in the study‐specific variance parameter $\sigma_{i}^{2}$. Note that β and γ are assumed common across studies, while for δ we assumed random effects. We can make alternative modeling choices, for example, to assume a common $\sigma^{2}$ for all trials, to assume random effects for β and γ, to assume exchangeability on the different γ for comparisons of active vs control and so forth. Also note, that the model above is a “one‐stage” model, that is, it performs a joint analysis of all data. Alternatively, we could follow a “two‐stage” approach,^18^ where the studies are analyzed on the first stage, and their results are meta‐analyzed on the second stage. We do not discuss such extensions further in this article.

### 3.2 Additive aggregate data component NMA

This model was originally presented by Welton et al in their seminal paper.^5^ Assume that study i compares treatment X, comprising components $c_{1}$ and $c_{2}$, vs treatment Y, comprising component $c_{3}$ and $c_{4}$. Also assume that for this study aggregate data (AD) are available, that is, relative treatment effects $y_{i}$ and variance $s_{i}^{2}$. The model is:

**Model III: AD additive CNMA (continuous outcome)**

$$y_{i}∼N\left(\delta_{i},s_{i}^{2}\right)$$

$$\delta_{i}∼N\left(\theta_{Y}-\theta_{X},\tau^{2}\right)$$

$$\theta_{X}=d_{1}+d_{2},\;\theta_{Y}=d_{3}+d_{4}$$

$$\tau^{2},d_{1},d_{2},\ldots ∼\left(\text{prior distributions}\right)$$

For multi‐arm trials we need multivariate normal distributions, as in Model I. Parameters $d_{q}$ ($q=1,2,\ldots N_{c},$ with $N_{c}$ being the total number of components) denote the benefit or harm of adding component $c_{q}$ to treatment. For example, when $y_{i}$ is mean difference, $d_{q}$ estimates the mean difference between treatments X and $X+d_{q}$, where X denotes any combination of components other than $c_{q}$, under the assumption of additivity. For the case of a binary outcome, we can use instead a binomial likelihood to model the probability of an event in each arm, for example, via a logit function. In this case, $d_{q}$ will denote the change in log‐odds when adding $c_{q}$ to any combination of components (ie, the log‐odds ratio between $c_{q}$ and $X+c_{q}$). Readers should note that $d_{q}$ are not estimates of absolute effect (eg, mean outcome or log odds), and should not be interpreted as such. The inputs of Model III (ie, $\delta_{i},s_{i}^{2}$) are on the relative effects level. Thus, our inference can be only about relative, not absolute, effects. This holds true for all models in this article, even models using arm‐level information (eg, events and non‐events per arm), because even in such cases we still synthesize relative effects (eg, log odds ratios) across trials. Also note that, depending on the network structure, some of the d parameters may be unidentifiable (eg, when $c_{1}$ and $c_{2}$ are always given together we cannot disentangle their effects). However, the relative effects between combinations of components compared in the trials will always be identifiable. We discuss issues regarding identifiability in more detail in Section 2 of the Appendix.

### 3.3 Aggregate data component NMA with component interactions

It is straightforward to add interactions between components to Model III.^5^, ^6^ For example, if we assume two‐way interactions only, the model can be written as follows:

**Model IV: AD‐CNMA with interactions (continuous outcome)**

$$y_{i}∼N\left(\delta_{i},s_{i}^{2}\right)$$

$$\delta_{i}∼\text{Normal}\left(\theta_{Y}-\theta_{X},\tau^{2}\right)$$

$$\theta_{X}=d_{1}+d_{2}+d_{1.2},\;\theta_{Y}=d_{3}+d_{4}+d_{3.4}$$

$$\tau^{2},d_{1},d_{2},\ldots ,d_{1.2},d_{1.3},\ldots ∼\left(\text{prior distributions}\right)$$

Parameter $d_{p.q}\;$ models the interaction between components $c_{p}$ and $c_{q}$. This can be positive, in which case the two components have synergistic effects, negative, in which case the effects are antagonistic, or zero, when the effects of the components are purely additive. Estimating an interaction term requires a specific network structure. For example, to estimate $d_{1.2}$ we need to have in the network studies including $c_{1}$ in some but not all of their treatment arms, studies including $c_{2}$, and studies including both $c_{1}$ and $c_{2}$ in the same arm (while also assuming that these effects can be disentangled from the rest of the components). Note that including too many interactions in the model will lead to some of the main or interaction terms to be unidentifiable. However, comparisons between combinations of components may still be identifiable, for example, an interaction model may be able to estimate $c_{1}+c_{2}$ vs $c_{3}$ without being able to separately estimate $d_{1},d_{2}$, $d_{3},$ and $d_{1.2}$. We discuss more about identifiability in Section 2 of the Appendix.

One question that comes up in practice is whether we should include interactions in the model and if yes, which ones. A priori, we may expect non‐zero interactions between any two components (or, to put it differently, it might be unrealistic to expect an interaction term to be *exactly* zero), although in practice the effects of these interactions might be negligible (they might be *practically* zero). The netcomb and discomb functions included in the **netmeta** package^7^ in R^19^ can be used for fitting frequentist CNMA models with interactions. Rücker et al^6^ recently described a method for deciding on whether the additive model or models with interactions provide better fit to the data, as compared to the standard NMA model. Rücker et al^9^ also described two stepwise model selection methods (a forward and a backward method) for deciding which interaction(s) to include in the model. These methods perform tests using Cochran's Q statistic to compare different models. A practical problem with this approach is that the number of tests can be large for networks with many components, comparing many different combinations. Moreover, stepwise variable selection methods in general have been criticized in the past,^20^ because they may lead to suboptimal model performance. We provide some additional detail on these issues in Section 3 of the Appendix.

### 3.4 Bayesian model selection in aggregate data component NMA with component interactions

We hereby propose a different way of addressing the question of which interactions to include in the model, utilizing Bayesian model selection methods. Such methods have been already shown to perform well for the case of estimating patient‐specific relative effects in pairwise IPD meta‐analysis.^21^ The main idea is that, instead of comparing all possible models with interactions with each other, we include all suspected interaction terms in the model, and shrink their corresponding coefficients. Coefficients of interactions for which there is small evidence in the data will be shrank more aggressively as compared to coefficients of interactions for which the data offers stronger evidence. Below we describe two different approaches.

We first discuss the method of stochastic search variable selection (SSVS). This Bayesian method is a form of the spike and slab model.^22^, ^23^ It was originally proposed by George and McCulloch^24^ and can be used to perform variable selection at each iteration of a Markov Chain Monte Carlo (MCMC) process. This is achieved through the introduction of indicator variables $I_{p.q}$, which, as we will see, have the advantage of allowing us to easily incorporate expert opinions. We hereby follow a variant of SSVS proposed by Meuwissen and Goddard^25^:

**Model V: AD‐CNMA with SSVS for interactions (continuous outcome)**

$$y_{i}∼N\left(\delta_{i},s_{i}^{2}\right)$$

$$\delta_{i}∼\text{Normal}\left(\theta_{Y}-\theta_{X},\tau^{2}\right)$$

$$\theta_{X}=d_{1}+d_{2}+d_{1.2},\;\theta_{Y}=d_{3}+d_{4}+d_{3.4}$$

$$\tau^{2},d_{1},d_{2},\ldots ∼\left(\text{prior distributions}\right)$$

$$\pi \left(d_{p.q}|I_{p.q}\right)=\left(1-I_{p.q}\right)\;N\left(0,\eta^{2}\right)+I_{p.q}\;N\left(0,g^{2}\eta^{2}\right)$$

η∼(narrowly distributed around zero),g=(large number)

$$I_{p.q}∼\left(\text{prior distribution}\right)$$

The idea behind this model is to have the prior distribution for the coefficients of the interaction terms we want to include in the model to be a mixture of two normal distributions. Both are centered around zero, but one is very narrow (“spike”), and one is vague (“slab”). When there is indication in the data that the interaction term between component $c_{p}$ and $c_{q}$ is non‐zero, then it is more likely for the interaction coefficient $d_{p.q}$ to have been derived from the vague normal distribution; conversely, if there is evidence that the value is close to zero, the narrow distribution is more likely. We denote by $\eta^{2}$ the variance of the narrow distribution, and $g^{2}$ is a large number multiplied to $\eta^{2}$, to create the variance of the vague distribution. Note here that the version of SSVS used in model V^25^ assumes a prior for η, for extra flexibility. Alternatively we can use the original version of SSVS^24^, which assumes a fixed value for η. The values of η will influence posterior estimates. η should take small values, but putting it too close to zero will make the SSVS model less efficient.^26^ Ideally, the choice of prior should be guided by practical considerations regarding the nature of the outcome. George and McCulloch^24^ mention that the choice for η should be such that for practical purposes the distribution $N\left(0,\eta^{2}\right)$ can be “safely” replaced by zero. For example, if a $d_{p.q}$ smaller than 0.5 is clinically unimportant, η could be given a prior $N\left(0,10^{-2}\right)$, because this distribution corresponds to interaction terms being practically zero. Given the prior for η, g should be large enough to allow for non‐zero values of $d_{p.q}$ to be supported. It should not be too large, however, because this way it would give support to unrealistic values.^24^ For example, if we are analyzing a binary outcome using log odds ratios or we are synthesizing log hazard ratios, a reasonable choice of priors might be $\eta ∼N\left(0,10^{-3}\right)$ and $g^{2}=100$.

The indicator variable $I_{p.q}$ shows which of the two normal distributions has been selected in each MCMC iteration. It can take values 0 or 1, and it controls whether $d_{p.q}$ will acquire a non‐zero value, or equivalently whether the interaction between $c_{p}$ and $c_{q}$ will be included in the model. Effectively, when $I_{p.q}=0$ the interaction term is absent from the model, and when $I_{p.q}=1$ it is included. The prior of $I_{p.q}$ reflects the prior probability for the interaction to be included in the model. If there is no prior knowledge about possible interactions between components, we could assign to $I_{p.q}$ a Bernoulli prior with probability 0.5. This choice, however, makes all models a priori equally probable, that is, it favors models that include half of the interactions. This might be unrealistic in most scenarios, and it might lead to identifiability issues. A much better option is to use expert opinion to first preselect interactions to be included in the model. All implausible interactions (eg, between components that cannot be combined, or between components for which there is prior knowledge that they do not strongly interact) should be excluded from the model upfront, by setting $d_{p.q}=0$. For the interactions to be included in the model, we can formulate informative priors for the $I_{p.q}$. For example, a very probable interaction may be given an 80% prior probability of being included in the model. This may be particularly useful when clinicians have insights about possible mechanisms of interactions between components. This feature constitutes an important advantage of SSVS.

One important characteristic of SSVS is that, strictly speaking, there is no overall, final variable selection. In other words, we do not end up with a single model. Rather, in each MCMC iteration we use a different model, as determined by the values for $I_{p.q}$ in that particular iteration (either 0 or 1). The importance of each interaction term in our final estimates can be assessed via the posterior distribution of $d_{p.q}\;\;$ and $I_{p.q}$. The latter tells us how often this interaction was included in the model. If for a particular interaction the data offers evidence that the effect is small, the corresponding parameter will shrink to zero, and it will have minimal effect on the model estimates. Note that we can easily include three‐way, four‐way, or higher‐order interactions in the model, following the exact same strategy. However, estimating such interactions in usual cases of data availability will be infeasible.

Another way to perform the analysis is to use a Laplacian shrinkage, also called a Bayesian LASSO^12^, ^27^ prior for $d_{c.c\prime }$. In this formulation, the interaction terms are assigned a Laplace (ie, double exponential) prior, which again tends to shrink their effects towards zero:

**Model VI: AD‐CNMA with Bayesian LASSO for interactions (continuous outcome)**

$$y_{i}∼N\left(\delta_{i},s_{i}^{2}\right)$$

$$\delta_{i}∼\text{Normal}\left(\theta_{Y}-\theta_{X},\tau^{2}\right)$$

$$\theta_{X}=d_{1}+d_{2}+d_{1.2},\;\theta_{Y}=d_{3}+d_{4}+d_{3.4}$$

$$\pi \left(d_{p.q}\right)=\frac{\lambda }{2}e^{-\lambda \left|d_{p.q}\right|}$$

$$\tau^{2},\lambda ,d_{1},d_{2},\ldots ∼\left(\text{prior distributions}\right)$$

The λ parameter of the Bayesian LASSO determines the amount of shrinkage performed to the $d_{p.q}$ parameters. For fitting purposes, we can treat λ as a random parameter and assign an informative hyperprior.^28^ In practice a sensitivity analysis might be required, while Lykou and Ntzoufras discussed strategies for constructing a prior for λ based on Bayes factors.^29^ Contrary to the frequentist LASSO (and similar to SSVS) this model does not perform any variable selection in the strict sense of the term. In other words, all interaction terms are included in the model, but their effects are shrunk towards zero. Interactions for which little evidence is available are shrunk more aggressively. Readers should note that it is much easier to include prior knowledge about interactions between components using SSVS, that is, via the indicator variables, rather than Bayesian LASSO. Also, it is not straightforward to select a prior for λ using expert opinion. Thus, in terms of applicability, SSVS has a clear advantage over Bayesian LASSO.

### 3.5 Individual patient data component NMA models

Let us now assume that some studies only provide aggregate data (“AD studies”), while some studies provide patient‐level data (“IPD studies”). The likelihood of our CNMA model now has two parts, one for each type of studies. Following the notation of the previous sections:

**Model VII: AD&IPD additive CNMA (continuous outcome)**

*Likelihood for an AD study comparing X vs Y*:

$$y_{i}∼N\left(\delta_{i},s_{i}^{2}\right)$$

*Likelihood for an IPD study comparing X vs Y*:

$$y_{\mathrm{ik}}∼N\left(\delta_{\mathrm{ik}},\sigma_{i}^{2}\right)$$

$$\delta_{\mathrm{ik}}=\left\{\begin{array}{c} \alpha_{i}+\beta^{\prime }x_{\mathrm{ik}}\;,\;\text{if}\;t_{\mathrm{ij}}=X=\left(c_{1}+c_{2}\right)\\ \alpha_{i}+\beta^{\prime }x_{\mathrm{ik}}+{\left(\gamma_{3}+\gamma_{4}-\gamma_{1}-\gamma_{2}\right)}^{\prime }x_{\mathrm{ik}}\;+\delta_{i\;},\;\text{if}\;t_{\mathrm{ij}}=Y=\left(c_{3}+c_{4}\right) \end{array}\right.$$

*Random effects structure and prior distributions*:

$$\delta_{i}∼N\left(\theta_{Y}-\theta_{X},\tau^{2}\right)$$

$$\theta_{X}=d_{1}+d_{2},\;\theta_{Y}=d_{3}+d_{4}$$

$$\tau^{2},d_{1},d_{2},\ldots \alpha_{l},\beta ,\gamma_{1},\gamma_{2},\ldots ∼\left(\text{prior distributions}\right)$$

Vector $\gamma_{q}$ includes the regression coefficients for effect modification (component‐covariate interaction), for component $c_{q}.$ It shows the added benefit of adding $c_{q}$ to the treatment, per unit increase of $x_{\mathrm{ik}}$. Component‐specific parameters $d_{q}$ are jointly estimated using the AD and IPD studies. Given the estimated parameters of this model, we can estimate the relative treatment effects between any two combinations of components for new patients given their covariates.

Of note, the IPD part of model VII provides conditional estimates of relative effects (ie, adjusted for covariates), while the AD part provides marginal estimates (ie, population average). At the second level of the model, these two estimates are pooled, that is, assuming they are exchangeable. We can expand the AD part by regressing over the mean value of the covariates in each study, for example, instead of $y_{i}∼N\left(\delta_{i},s_{i}^{2}\right)$ we use $y_{i}∼N\left(\delta_{i}+\beta \prime \bar{x_{i}},s_{i}^{2}\right)$.^30^, ^31^

Note that model VII requires the estimation of potentially many parameters. For example, for the depression case study there are 17 components and 4 covariates, which means that we need to estimate 68 different coefficients in γ. Aiming for a better generalizability of findings, and to avoid issues related to overfitting, we can also apply a shrinkage method for the γ. One way is to extend this model by using again SSVS or Bayesian LASSO for γ, that is, shrinking the coefficients towards zero. For a continuous outcome, we use a conditional Laplace prior on the γ, as proposed originally by Park and Casella.^28^ This has the added feature that it leads to unimodal posteriors for the coefficients. To use this prior we need to assume a common $\sigma_{i}^{2}$ for all IPD studies, that is, $\sigma_{i}^{2}=\sigma^{2}$. The prior is then:

$$\pi \left(\gamma^{\left(c\right)}|\sigma^{2}\right)=\prod_{k=1}^{N_{c}}\frac{\lambda }{2\sigma }e^{-\lambda \;\left|\gamma_{k}^{\left(c\right)}\right|/\sqrt{\sigma^{2}}}$$

where $N_{c}$ is the number of covariates. Note that this form of the Bayesian LASSO could not be used for aggregate data, for example, in Model VII. The reason is that we cannot assume a single $\sigma^{2}$, because each study estimates comes with each own precision, $s_{i}^{2}$.

Model VII can be further extended to also include interactions between components:

**Model VIII: AD&IPD CNMA with component interactions (continuous outcome)**

*Likelihood for an AD comparing X vs Y*:

$$y_{i}∼N\left(\delta_{i},s_{i}^{2}\right)$$

*Likelihood for an IPD comparing X vs Y*:

$$y_{\mathrm{ik}}∼N\left(\delta_{\mathrm{ik}},\sigma_{i}^{2}\right)$$

$$\delta_{\mathrm{ik}}=\left\{\begin{array}{c} \alpha_{i}+\beta^{\prime }x_{\mathrm{ik}}\;,\;\text{if}\;t_{\mathrm{ij}}=X=\left(c_{1}+c_{2}\right)\\ \alpha_{i}+\beta^{\prime }x_{\mathrm{ik}}+{\left(\gamma_{3}+\gamma_{4}-\gamma_{1}-\gamma_{2}\right)}^{\prime }x_{\mathrm{ik}}\;+\delta_{i\;},\;\text{if}\;t_{\mathrm{ij}}=Y=\left(c_{3}+c_{4}\right) \end{array}\right.$$

*Random effects, component specification and prior distributions*:

$$\delta_{i}∼N\left(\theta_{\Upsilon }-\theta_{\mathrm{X;}},\tau^{2}\right)$$

$$\theta_{X}=d_{1}+d_{2}+d_{1.2},\;\theta_{Y}=d_{3}+d_{4}+d_{3.4}$$

$$\tau^{2},d_{1},d_{2},\ldots \alpha_{i},\beta ,\ldots ∼\left(\text{prior distributions}\right)$$

$$d_{p.q}∼\left(\text{SSVS or Bayesian LASSO prior distribution}\right)$$

$$\gamma_{1},\gamma_{2},\ldots ∼\left(\text{SSVS or Bayesian LASSO prior distribution}\right)$$

This model can be easily modified to address more complex modeling requirements, for example, to include a richer random effect structure (eg, on the β or γ parameters) or to assume exchangeability on the component effects.^32^ Binary outcomes and multi‐arm studies can be accounted for as discussed in previous sections.

### 3.6 Utilizing an IPD‐NMA model in clinical practice

An IPD‐NMA model (such as Model II) can estimate relative treatment effects for every treatment comparison and for any combination of patient covariates. Furthermore, IPD‐CNMA models (such as Models VII and VIII) have the capacity to do so for any combination of components. Thus, such models could be used to inform the choice of treatments in clinical practice at the patient level; this might be particularly important in some medical fields, where assigning a patient to an intervention is often a matter of trial and error. In practice, this requires some sort of calculator, where a user can input patient characteristics and obtain an estimate of treatment effects. In Section 4 of the Appendix we provide some details on developing an online web application using **shiny** ^33^ in R. We describe such an app in the results, Section 4.2.

### 3.7 A note on imputing missing covariate data in IPD studies

One usual problem in IPD studies is missing covariate data. Missing data can be either systematically missing (eg, when a study does not collect information on patients' relationship status), or sporadically missing (eg, when some patients did not provide information about their age). To use the IPD models described in previous sections we need to impute missing covariates, otherwise patients with incomplete covariate data will be excluded from the analyses. For that, we follow the recommendations by Zhou and Reiter^34^: we create multiply imputed datasets, we fit the IPD models in each imputed dataset separately, and we mix the posterior draws, to summarize the posterior distribution.

## 4 APPLICATIONS

Here we give results from the analysis of the datasets presented in Section 2. The R code used for all analyses is available in https://github.com/esm‐ispm‐unibe‐ch/Bayesian‐CNMA.

### 4.1 Panic disorder

This dataset had only aggregate data. Each study provided the number of events and number of patients per treatment arm. For the analysis we employed four different models: (i) Model III, the additive AD‐CNMA model assuming no component interactions; (ii) Model V, the AD‐CNMA model with SSVS for interactions. We assumed equiprobable interactions, that is, for all $I_{p.q}$ we assumed $I_{p.q}∼\text{Bernoulli}\left(0.5\right)$, and we used $\eta ∼N\left(0,10^{-2}\right)$ and g=100; (iii) Model V again, using clinical opinion^4^ to inform the probability of inclusion for some interaction terms. More specifically, for six interactions we assumed $I_{p.q}$ to follow a Bernoulli distribution with p=0.8: ftf + ine, ftf + cr, pe + ine, cr + ive, br + ine, br + ive. Other interactions were excluded from the model; (iv) Model VI using a Bayesian LASSO prior for all interactions. For the penalization parameter of the model, we used $\lambda^{-1}∼U\left(0,5\right)$ as a prior distribution. For all analyses we used Binomial likelihood at the arm level. We assumed an informative prior distribution for $\tau^{2}$, namely $\tau^{2}∼\mathrm{LN}\left(-1.67,1.472^{2}\right)$, based on empirical data,^35^ vague priors for the baseline log‐odds (N(0,100)), and vague priors for the main component effects, $d_{q}∼N\left(0,10^{2}\right)$ for all q. For all models, we estimated the log odds ratio for each component, and heterogeneity SD (τ). Also, for illustration purposes we estimated the odds ratios for the following comparisons across all models: (pl + ftf + pe + ps + ive) vs (wl); (pl + ftf + pe + ps + ive + cr) vs (pl + ftf + ps + mr); (pf + ftf + ps + ive + cr) vs (pl + pe + br + mr + ine + cr). We used four independent chains of 30 000 MCMC iterations after 10 000 iterations burn‐in and we checked convergence by visually checking the posteriors and the mixing of the chains. The analyses took 30 min to run on a laptop computer.

**FIGURE 1:**
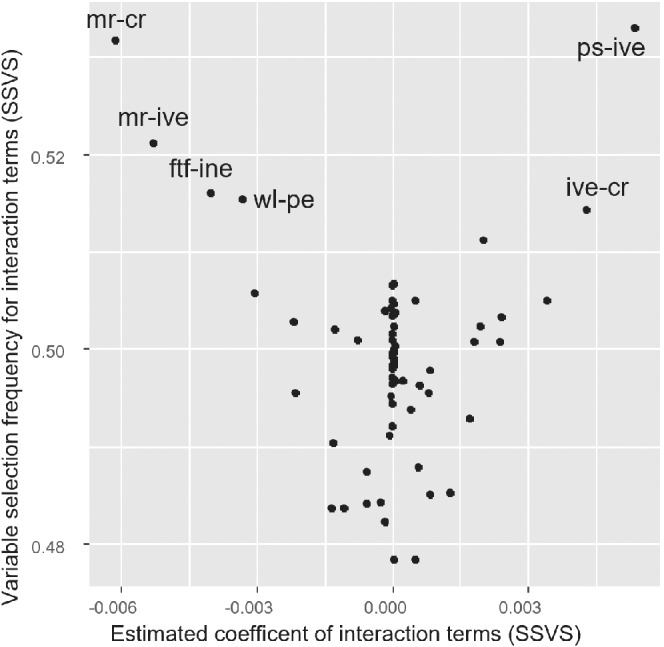
Results for component interactions from the stochastic search variable selection (SSVS) model for the panic disorder example, where all interactions were assumed equiprobable. *x*‐axis: estimated interaction terms (log odds ratios); *y*‐axis: frequency of selection for each interaction term (ie, percent of times the interaction term was included in the model). The six most prominent interactions are labeled. Component abbreviations given in Section 2.1

In Table 1 we show results. We present the estimated d parameter for each component (in log‐odds ratios), heterogeneity τ, and estimated effects for the treatment comparisons defined above, in odds ratios. We saw that the inclusion of interaction terms slightly reduced the estimated heterogeneity as compared to the additive model, suggesting that interactions may explain some of the observed variation. In Figure 1 we show the estimated point estimates of all interaction terms vs their inclusion frequency for the SSVS model with equiprobable interactions. We see a roughly V‐shaped distribution, as expected: interaction terms with larger estimated absolute values had a higher probability of being included in the model. Interestingly, two of the interactions that stand out (ftf‐ine and ive‐cr) were among the six suggested by the experts. Note that many points are in the middle of the plot, that is, with a coefficient around zero and inclusion probability around 50%. These were interaction terms for which there was small information in the data. Aiming to assess the sensitivity of our results on the choice of priors, we repeated analyses using different priors for main effects, that is, $d_{q}$∼U(−3,3), different priors for the baseline log‐odds, that is, U(−3,3). We also tried a different prior for λ in model (iv), that is, $\lambda^{-1}∼N\left(0,100\right)I\left(0,\right)$. In all cases, we saw small differences in results, which did not materially change our conclusions.

**TABLE 1:** Estimated log odds ratios [95% credible intervals] for each component (wl, pl, …, w3), heterogeneity (τ), and odds ratios for three treatment comparisons, based on three different CNMA models

Estimated quantity	Additive CNMA (no interactions)	CNMA with SSVS (equiprobable interactions)	CNMA with SSVS (external information for interactions)	CNMA with Bayesian LASSO
wl	−2.64 [−4.11; −1.26]	−2.66 [−4.20; −1.26]	−2.57 [−4.06; −1.18]	−2.75 [−4.63; −1.17]
pl	0.17 [−1.16; 1.52]	0.23 [−1.13; 1.68]	0.20 [−1.14; 1.53]	0.30 [−1.20; 1.98]
ftf	−0.11 [−1.09; 0.88]	−0.15 [−1.44; 1.07]	−0.05 [−1.13; 1.08]	−0.23 [−2.22; 1.40]
pe	−0.30 [−1.11; 0.46]	−0.36 [−1.49; 0.59]	−0.29 [−1.11; 0.48]	−0.46 [−2.20; 0.75]
ps	0.00 [−1.09; 1.10]	−0.08 [−1.36; 1.10]	0.00 [−1.12; 1.10]	−0.20 [−1.80; 1.13]
br	−0.13 [−0.71; 0.42]	−0.12 [−0.99; 0.79]	−0.13 [−0.79; 0.52]	−0.13 [−1.51; 1.30]
mr	−0.62 [−1.18; −0.07]	−0.50 [−1.30; 0.61]	−0.63 [−1.20; −0.07]	−0.38 [−1.54; 1.31]
ive	−0.19 [−0.77; 0.43]	−0.32 [−1.57; 0.52]	−0.26 [−0.96; 0.41]	−0.53 [−2.77; 0.59]
ine	0.29 [−0.35; 0.92]	0.36 [−0.50; 1.45]	0.39 [−0.39; 1.36]	0.49 [−0.70; 2.20]
vre	−0.10 [−1.44; 1.30]	−0.11 [−1.73; 1.51]	−0.08 [−1.42; 1.34]	−0.17 [−2.35; 2.00]
cr	0.31 [−0.28; 0.92]	0.23 [−0.82; 1.08]	0.25 [−0.58; 0.98]	0.12 [−1.68; 1.30]
w3	−0.26 [−2.69; 2.15]	−0.15 [−2.69; 2.41]	−0.16 [−2.59; 2.21]	0.02 [−2.84; 3.08]
τ (heterogeneity SD)	0.66 [0.28; 1.37]	0.61 [0.23; 1.33]	0.64 [0.25; 1.34]	0.56 [0.16; 1.26]
Estimated odds ratios for example comparisons				
(pl + ftf + pe + ps + ive) VS (wl)	9.15 [4.49; 20.35]	8.92 [4.26; 20.22]	8.62 [4.19; 19.60]	8.88 [4.22; 20.34]
(pl + ftf + pe + ps + ive + cr) VS (pl + ftf + ps + mr)	1.55 [0.55; 4.53]	1.51 [0.51; 4.55]	1.54 [0.54; 4.46]	1.51 [0.48; 4.86]
(pf + ftf + ive + cr) VS (pl + pe + br + mr + ine + cr)	1.60 [0.29; 9.78]	1.23 [0.15; 9.26]	1.59 [0.29; 10.39]	0.90 [0.05; 9.52]

*Note*: Abbreviations of components in Section 2 of this article.

Abbreviations: CNMA, component network meta‐analysis; SSVS, stochastic search variable selection.

In general, we saw some discrepancies in the estimated effects across models, especially when comparing the Bayesian LASSO with the additive model. However, differences were not clinically important. In summary, we conclude that, there is only weak evidence of some relatively small interactions between components for this example.

### 4.2 Depression

This dataset included both AD and IPD studies. To account for missing data in the IPD studies, we created multiply imputed datasets (m=10) with the **jomo** package^36^ in R^19^ and used Rubin's rules to estimate aggregated treatment effects from each study separately. Then we fit three models: (i) Model III (ii) Model IV with equiprobable interactions, where, assuming values of the outcome smaller than 0.5 to be clinically unimportant we used $\eta ∼N\left(0,10^{-2}\right)$ and g=100; (iii) Model VI. In all models we assumed a normal likelihood at the arm level, with a prior for baseline outcome N(0,100). Heterogeneity was given a vague prior, τ∼N(0,10)I(0,). Other model details were as described in the previous paragraph. For illustration purposes, we estimated the relative treatment effects for the following comparisons: (pl + cr + ba +ps) vs (wl); (pl + w3 + ftf) vs (wl); (pl + pe + ae) vs (wl). Note that the drug therapy component (dt) was either present or absent in all arms of the included studies; thus, the corresponding d parameter was not estimable. However, its interactions with other components were included in the interaction models. Analyses used four independent chains, 30 000 iterations after 10 000 iterations burn‐in, convergence assessed as above. The analyses took approximately 30 minutes to run in a laptop computer.

**FIGURE 2:**
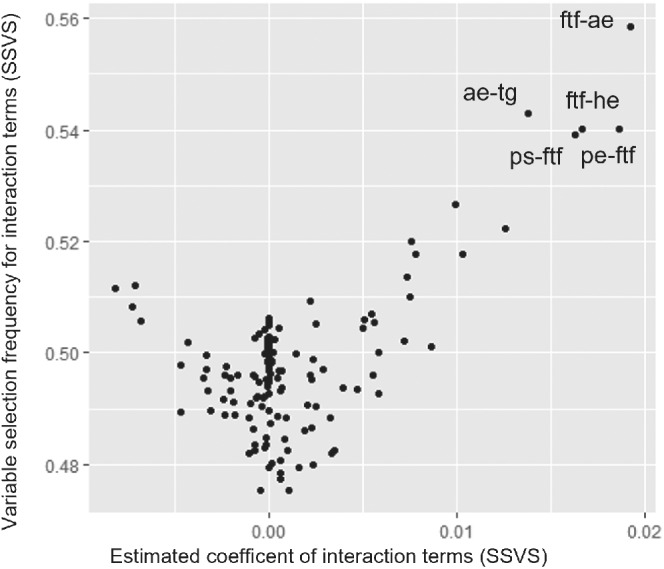
Estimated interaction terms (in PHQ‐9) for components of psychotherapies for depression on the *x*‐axis, and corresponding indicator variables (ie, probability of being included in the model) on the *y*‐axis. The five most prominent interactions are labeled in the graph. Component abbreviations given in Section 2.2

Results are shown in Table 2. As in the previous example, results from the SSVS and Bayesian LASSO CNMA models with interactions were almost identical. Compared to the additive model, it is obvious that the inclusion of interactions had a rather small impact on the estimate for τ, suggesting that interactions terms were not able to explain much of the observed heterogeneity between the studies. The estimates for the components were slightly different in some cases, but the three estimated relative treatment effects were rather similar across all three models. Figure 2 shows the scatterplot of the indicator variables vs estimated interaction effects from SSVS. The interactions with the highest inclusion probability are labeled on the graph and were mostly related to the face‐to‐face (ftf) component. The same interactions were also picked up by the Bayesian LASSO. However, the corresponding coefficients were very small, bearing little evidence of clinically important interactions. We repeated the analysis using a different vague prior for d∼U(−5,5). Also, using different priors for the baseline outcome (U(0,30]). We also tried a different prior for heterogeneity, τ∼U(0,5) and a different prior for the Bayesian LASSO, $\lambda^{-1}∼N\left(0,100\right)I\left(0,\right)$. In all cases results did not materially change.

**TABLE 2:** Estimated values [95% credible intervals] for each component (wl, pl, …, tg), heterogeneity parameter (τ), and relative effects (mean differences in PHQ‐9) for three treatment comparisons, based on three different CNMA models

Estimated quantity	Additive CNMA (no interactions)	CNMA with SSVS (equiprobable interactions)	CNMA with Bayesian LASSO
wl	0.17 [−1.00; 1.34]	0.24 [−1.08; 1.54]	0.24 [−1.14; 1.56]
dt	0.11[−61.99;61.93]	0.05[−62.11;61.16]	0.04[−19.48;19.53]
pl	−1.56 [−2.73;‐0.38]	−1.30 [−2.61; 0.08]	−1.28 [−2.63; 0.15]
pe	0.06 [−0.88; 1.01]	−0.18 [−1.50; 0.91]	−0.21 [−1.62; 0.92]
cr	0.40 [−0.78; 1.55]	0.19 [−1.48; 1.60]	0.17 [−1.59; 1.60]
ba	−1.92 [−3.03;‐0.84]	−1.75 [−3.12;‐0.23]	−1.75 [−3.17;‐0.20]
is	−0.55 [−1.61; 0.54]	−0.48 [−2.06; 1.25]	−0.49 [−2.16; 1.45]
ps	−0.64 [−1.44; 0.15]	−0.62 [−1.95; 0.78]	−0.59 [−2.08; 0.99]
re	1.05 [−0.01; 2.13]	0.98 [−0.70; 2.55]	0.97 [−0.85; 2.56]
w3	−0.60 [−1.69; 0.47]	−0.85 [−2.57; 0.51]	−0.89 [−2.69; 0.50]
bi	−2.19 [−4.35; 0.02]	−2.00 [−4.54; 0.68]	−1.98 [−4.54; 0.81]
rp	0.22 [−0.84; 1.25]	0.32 [−1.04; 1.79]	0.37 [−1.08; 1.91]
hw	0.24 [−0.82; 1.34]	0.39 [−0.98; 2.06]	0.40 [−1.05; 2.17]
ff	0.76 [−2.22; 3.73]	0.59 [−2.44; 3.64]	0.60 [−2.43; 3.62]
ae	−0.29 [−1.21; 0.61]	−0.39 [−1.92; 0.88]	−0.44 [−2.25; 0.91]
he	−0.32 [−1.30; 0.65]	−0.33 [−1.66; 1.08]	−0.33 [−1.75; 1.14]
tg	0.17 [−0.79; 1.15]	0.00 [−1.77; 1.43]	−0.05 [−2.01; 1.43]
τ (heterogeneity SD)	1.31 [0.99; 1.71]	1.23 [0.85; 1.65]	1.22 [0.83; 1.64]
*(pl + cr + ba + ps)* VS *(wl)*	−3.87 [−5.58;‐2.28]	−3.75 [−5.71;‐1.80]	−3.74 [−5.74;‐1.69]
*(pl + w3 + ftf)* VS *(wl)*	−1.58 [−4.72; 1.55]	−1.78 [−5.11; 1.53]	−1.80 [−5.16; 1.49]
*(pl + pe + ae)* VS *(wl)*	−1.96 [−3.28;‐0.64]	−2.11 [−4.06;‐0.44]	−2.17 [−4.28;‐0.41]

*Note*: Abbreviations of components in Section 2 of this article.

Abbreviations: CNMA, component network meta‐analysis; SSVS, stochastic search variable selection.

Next, we fitted an IPD‐CNMA model in the data. Based on the results from the analysis at the aggregate level presented above we decided to not include interactions between components, to keep the model relatively simple. Thus, we fitted Model VIII with a Bayesian LASSO to model the component‐covariate interactions. For this, we used multiple imputation with 10 repetitions, and we fit the IPD‐CNMA model in each multiply imputed dataset separately. We used a single chain, of 1000 iterations after 500 burn‐in. At the end we mixed the draws from the 10 imputed datasets, to create the final posterior distribution.^34^ The analysis took approximately 30 hours to run in a laptop computer. Results for all model parameters are given in Section 2 of the Appendix. Heterogeneity was only slightly decreased and was estimated 1.20 [0.89; 1.57], that is, only a small part of the heterogeneity was explained by inclusion of patient‐level information from the IPD studies. Overall, there was evidence that baseline severity was strongly prognostic. Age and relationship status were found to be less important prognostic factors, while there was no evidence for a prognostic role of gender. The effect modification due to covariates (ie, component‐covariate interactions) was stronger for baseline severity as compared to age, gender or relationship status, but effects were generally small. The estimated effects of the components were comparable to the ones presented in Table 2, showing strong evidence for beneficial effects of two components (pl, ba) and strong evidence for a detrimental effect of one component (re).

**FIGURE 3:**
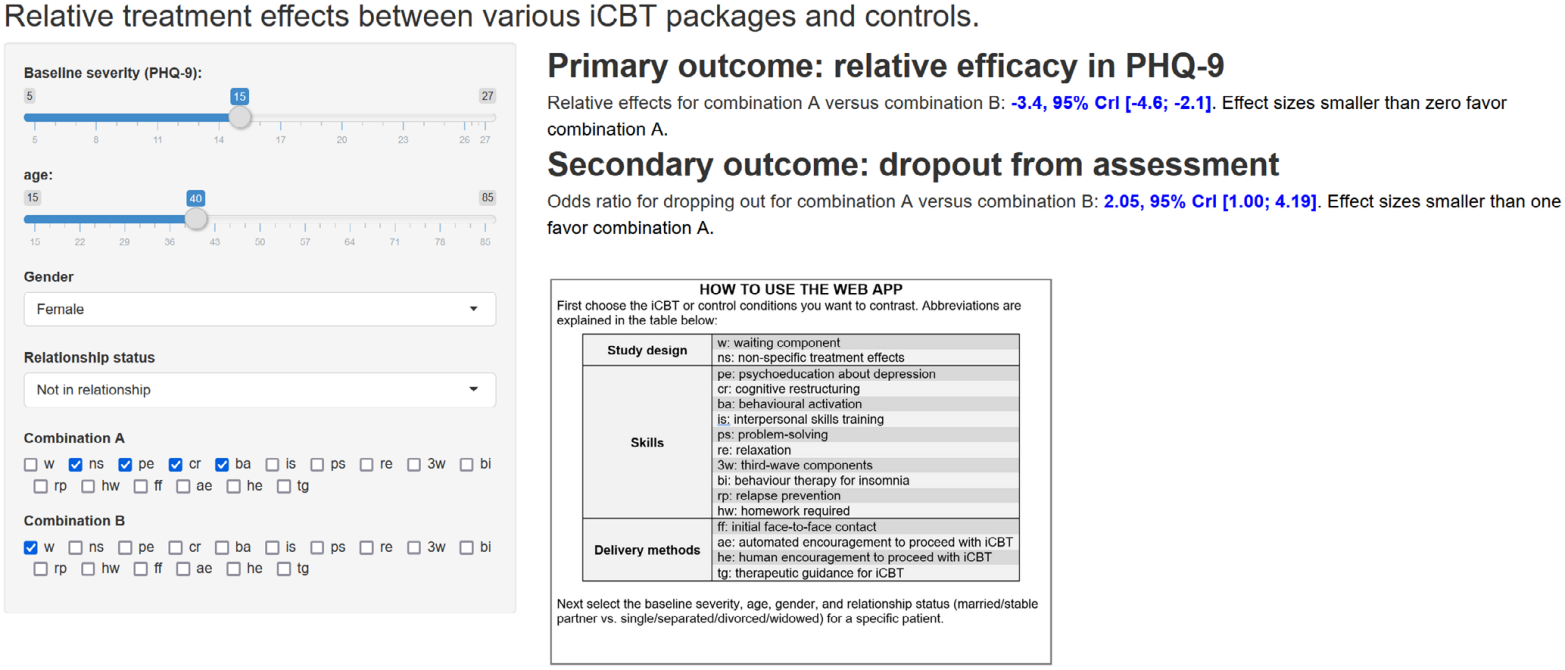
Snapshot of the web application for utilizing the IPD component NMA model in clinical practice. The user inputs patient characteristics and combination of components to be compared. The app provides the estimated relative treatment effects for the two combinations, for two outcomes of interest

Based on the results of this model we developed a web‐application^15^ accessible in https://esm.ispm.unibe.ch/shinies/cNMA_iCBT/. A snapshot of the web‐app is shown on Figure 3. This app can be used to input patient characteristics (ie, baseline severity, age, gender, and relationship status) and two combinations A and B of components (left panel). Outputs of the app are estimated treatment effects and 95% Credible Intervals among combinations A and B (right panel).

## 5 DISCUSSION

We presented a set of models for performing Bayesian CNMA. One difficulty when using CNMA is deciding which interactions of components to include in the model, if any. Here, we illustrated the use of Bayesian variable selection methods for identifying the most prominent interactions. More specifically, we used the SSVS and the Bayesian LASSO methods. Both tend to shrink the effect of interaction terms; for interactions for which the data suggests a weak effect, the corresponding parameters will shrink close to zero and have a minimal impact on the estimated relative treatment effects. For interactions for which there is evidence of an effect, the shrinkage will be much less. The advantage of this approach, as compared to a previously published method for selecting interactions,^9^ is that it avoids possible deficiencies associated with stepwise selection. Moreover, the use of Bayesian methods, and especially SSVS, conveys the advantage of facilitating the incorporation of external (prior) information about important interactions, as well as about other parameters of the model that may be hard to estimate, such as heterogeneity. In addition, our Bayesian methods offer increased flexibility in model building, such as the potential to use binomial likelihood for binary outcomes, specify random effects structures and so forth. Among the two variable selection models, we promote the use of SSVS, because it is much easier to decide on prior distributions for parameters as compared to Bayesian LASSO, when incorporating expert opinion. One limitation of both approaches is that they assume familiarity with Bayesian methods and software, and require a careful selection of the priors for all model parameters. This is especially true for priors for interactions between components, because in practice there may be weak information in the data regarding most of them. Notably, in practical applications, some component interactions may be impossible to exist, for example, between mutually exclusive components. In general, it is good practice to pre‐select interactions based on clinical opinion, and exclude from the model such impossible interactions, interactions that are improbable, or interactions that are expected to be very weak according to experts. We hereby illustrated these methods in three real examples; in all examples the inclusion of interactions did not materially change results, as compared to the simple additive model. This suggests that the additivity assumption may be a good approximation for many cases in practice. However, more empirical evidence is needed to clarify this issue.

In this article we also described how to generalize CNMA models to allow the inclusion of IPD from all or only some studies in the network. We demonstrated how IPD CNMA models can include interactions between components, but also interactions between covariates and components. The latter goes into the direction of personalized (“stratified”) medicine, that is, when the estimated treatment effect depends on the patient characteristics. We discussed how to develop online calculators that can facilitate their use, that is, to take full advantage of such analyses in clinical practice. One limitation is that the IPD‐NMA models we presented did not differentiate the within‐ from the across‐study interactions of the covariates and the components.^16^ However, they could be modified to accommodate such a change.

There are several other potential extensions of the work described in this article. First, we could envision models that also shrink the main effects of the components, and not just their interactions. This would be straightforward, using either LASSO or SSVS. However, with SSVS we may end up with “unnatural” models in each MCMC iteration, where the main effect of a component is excluded from the model while some of its interactions are included. Whether this would lead to better or worse estimates of relative treatment effects is unclear. Related to this, an interesting area of future work is to perform a simulation study to explore the advantages and disadvantages of the Bayesian models and to see how these compare to frequentist approaches.

To summarize, we have presented a range of Bayesian models for CNMA. Our models facilitate the identification of important component interactions and can combine aggregate and patient‐level data to estimate patient‐specific relative treatment effects.

## Supporting information


**Appendix S1**: Supporting Information

## Data Availability

This dataset has been described in more detail elsewhere. We show the network in the Appendix, and we provide the data in GitHub. Due to restrictions in data sharing agreements, the data cannot be made publicly available.
