# Supplementary material for: Bayesian models for aggregate and individual patient data component network meta‐analysis
Source: Stat Med. 2022 Mar 8;41(14):2586–601. doi: 10.1002/sim.9372 (PMC9314605; doi:10.1002/sim.9372)
Supplement: Supplementary file 1 — Appendix S1: Supporting Information [file SIM-41-2586-s001.docx]

**Supplementary material for “Bayesian models for aggregate and individual patient data component network meta-analysis”**

Orestis Efthimiou^1,2^, Michael Seo^1,3^, Eirini Karyotaki^4,5,6^, Pim Cuijpers^5^, Toshi Furukawa^7^, Guido Schwarzer^8^, Gerta Rücker^8^, Dimitris Mavridis^9,10^

*^1^Institute of Social and Preventive Medicine, University of Bern, Switzerland*

*^2^Department of Psychiatry, University of Oxford, Oxford, United Kingdom*

*^3^ Graduate School for Health Sciences, University of Bern, Bern, Switzerland*

*^4^Department of Global Health and Social Medicine, Harvard Medical School, Boston, USA*

*^5^Department of Clinical Neuro- and Developmental Psychology, Vrije Universiteit Amsterdam, the Netherlands*

*^6^Amsterdam Public Health Research Institute, Amsterdam, the Netherlands*

*^7^Departments of Health Promotion and Human Behavior and of Clinical Epidemiology, Kyoto University Graduate School of Medicine/School of Public Health, Kyoto, Japan*

*^8^Institute of Medical Biometry and Statistics, Faculty of Medicine and Medical Center –University of Freiburg, Freiburg, Germany*

*^9^Department of Primary Education, University of Ioannina, Ioannina, Greece*

*^10^Faculté de Médecine, Université Paris Descartes, Paris, France*

# **Network graphs for the clinical examples used in the paper**

## *Treatments for panic disorder*

The network at the treatment level is shown in Figure 1. Numbers on edges indicate number of studies performing the corresponding comparison. Thickness of edges indicates the precision of estimated relative effects from a network meta-analysis. Abbreviations: Waiting list (WL), No treatment (NT), Supportive psychotherapy (SP), Face-to-face physiological therapy (PT), Face-to-face cognitive therapy (CT), Self-help behaviour therapy (SH-BT), Face-to-face behaviour therapy (BT), Self-help cognitive-behaviour therapy (SH-CBT), Face-to-face cognitive-behaviour therapy (CBT), Self-help third wave CBT (SH-3rd Wave), Self-help physiological therapy (SH-PT).

These treatments were combinations of several components: wl (watiting list), pl (placebo) ftf (face-to-face), pe (psychoeducation), ps (psychological support), br (breathing retaining), mr (progressive/applied muscle relaxation), ive (in vivo exposure), ine (interoceptive exposure), vre (virtual reality exposure), cr (cognitive restructuring) and w3 (third wave). The definition of treatment nodes was given in Table 2 of the original publication^1^. For instance the combination pl + ps + ftf with or without pe was defined to be SP (Supportive psychotherapy). Following these definitions, there might have been studies comparing different combinations of components which might fall into the same treatment node. For example, a three-arm study might be comparing no treatment (NT) versus pl + ps + ftf (which according to the definition above is SP) versus pl + ps + ftf + pe (which is also SP). This study has therefore 1 NT arm and 2 SP arms. In this case, the two different SP arms of the study would need to be merged for the analysis at the treatment level, and subsequently for plotting the network graph. If there were studies where all treatment arms fell under the same treatment node (e.g. a study comparing pl + ps + ftf versus pl + ps + ftf + pe, i.e. SP versus SP), these studies would need to be excluded from the analysis at the treatment level – although they can still be analysed in the component NMA.

At the component level the network is disconnected in three subnetworks. The complete list of comparisons among combinations is depicted in Figure 2.


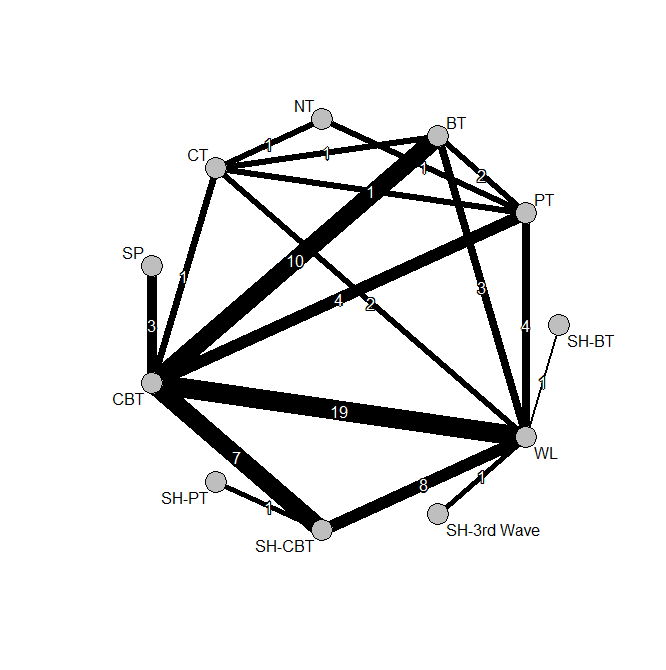


**Figure 1:** Network at the treatment level for panic disorder studies


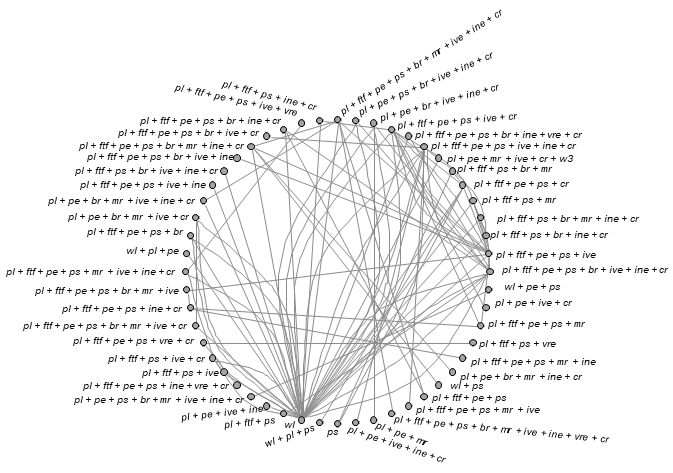


**Figure 2:** Network at the component level for panic disorder studies.

## *Treatments for depression*

The network at the treatment level is shown in **Figure 3**. Numbers on edges indicate number of studies performing the corresponding comparison. Thickness of edges indicates the precision of estimated relative effects from a network meta-analysis. Abbreviations: Waiting list (WL), No treatment (NT), Face-to-face physiological therapy (PT), Face-to-face cognitive therapy (CT), Face-to-face cognitive-behaviour therapy (CBT), Third Wave (3W) Treatment as usual (TAU), Psychoeducation (PE), Attention Placebo (APP).


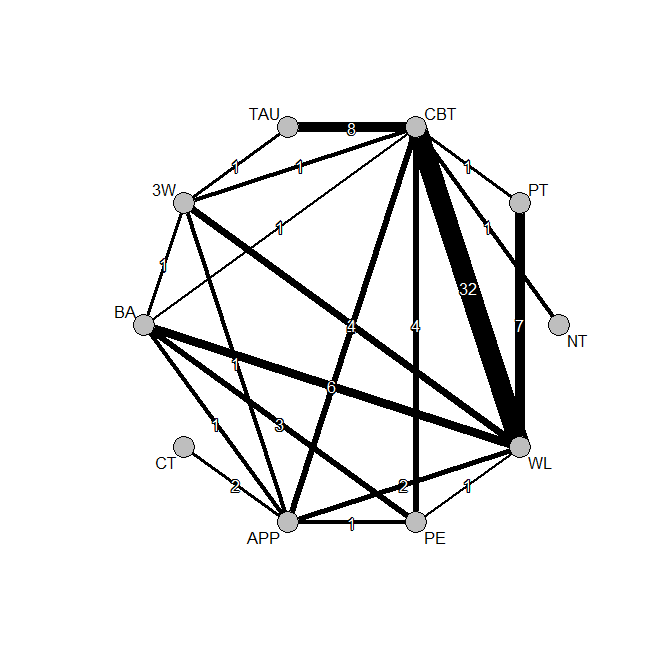


**Figure 3:** Network at the treatment level for depression studies.

These ten treatments were in fact a combination of 17 components: wl (waiting list), dt (drug therapy), pl (placebo), pe (psychoeducation), cr (cognitive restructuring), ba (behavioural activation), is (interpersonal skill trainint), ps (problem solving), re (relaxation), w3 (third wave), bi (behavioural therapy for insomnia), rp (relapse prevention), hw (homework required), ftf (face to face), ae (automated encouragement), he (human encouragement) and tg (therapeutic guidance). The network at the component level (i.e. assuming each component as a separate treatment) is depicted in **Figure 4** . This is not connected, it comprises multiple disconnected subnetworks.


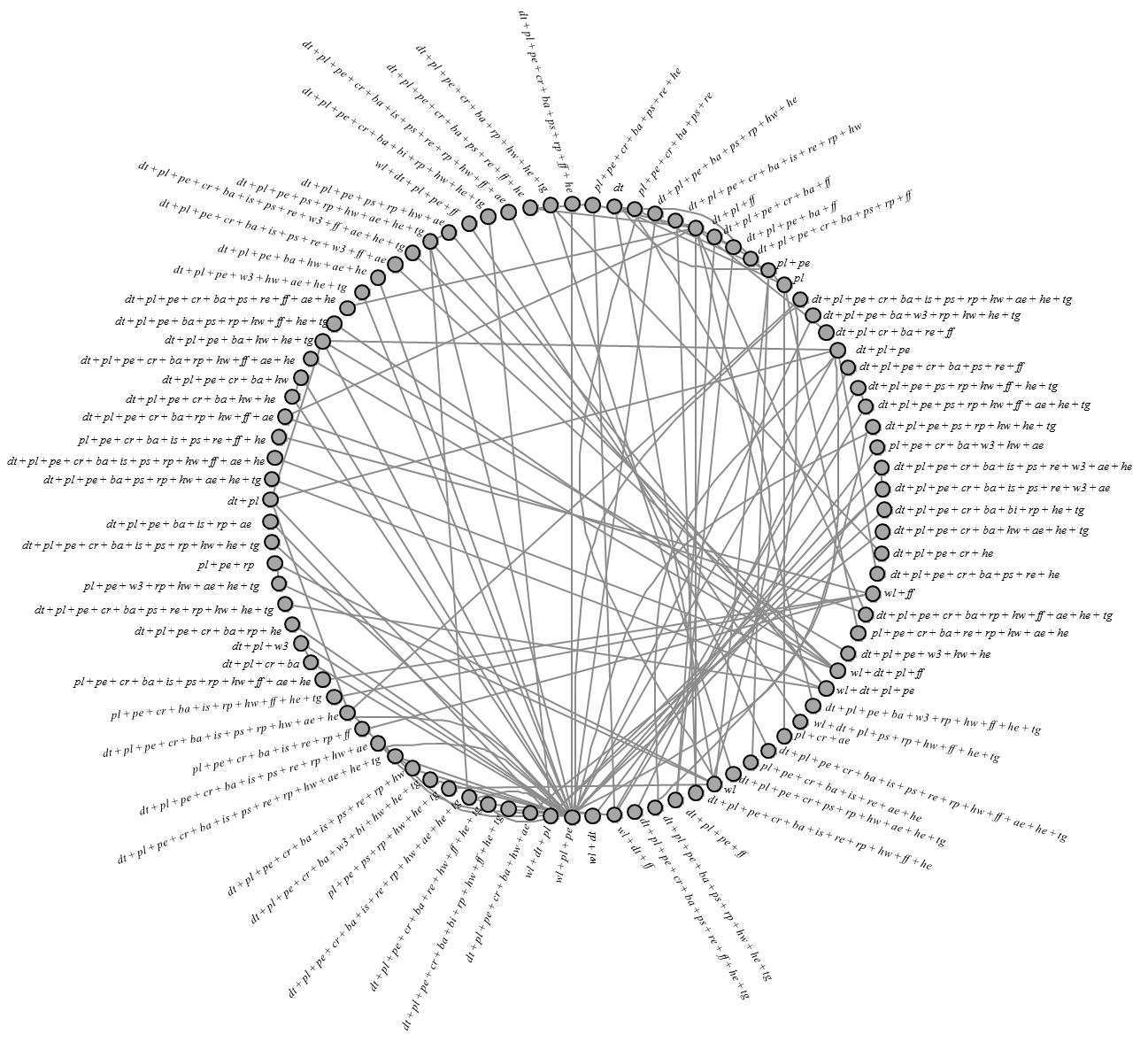


**Figure 4:** Network at the component level for depression studies.

# **Issues related to parameter identifiability**

In this Section, we clarify through theoretical arguments and simple examples issues that come up when some of the parameters of a component NMA model are unidentifiable.

## *Component NMA model without interactions*

Here we discuss about additive CNMA models, i.e. no interactions between components. Let us assume that we have 4 components, and only 3 studies in the network.

- Study 1 compares c1+c2 vs. c2. Under additivity, this study provides information on $d_{1}$.
- Study 2 compares c3+c4 vs. c4. Under additivity, this study provides information on $d_{3}$.
- Study 3 compares c1+c2 vs. c3+c4. Under the additivity assumption (i.e. no interactions), this study provides information on $d_{1}+d_{2}-d_{3}-d_{4}$.

Overall, assuming additivity, this network allows to estimate only the following relative treatment effects:

- c1+X vs X (where X is any combination of components, e.g. X=c2, or X=c2+c3, etc., because X cancels out)
- c3+X vs X (where X is any combination of components, because X cancels out)
- c1+c2 vs. c3+c4, because of study 3.

In a frequentist setting we would say that $d_{1}$ and $d_{3}$ are identifiable but $d_{2}$ is not, i.e. it is easy to see that we cannot estimate the effect of component c2 from this dataset. However, although $d_{2}$ is unidentifiable, $d_{1}+d_{2}-d_{3}-d_{4}$ (which includes $d_{2}$) is identifiable, due to study 3.

Let us now consider the case of a Bayesian NMA model with flat priors for all $d$ parameters, and no interaction terms. Of course, the model has priors for all parameters, even $d_{2}$ (which we said is “unidentifiable”), meaning that after fitting the model we obtain a posterior $d_{2}$. This also holds for comparisons that were unidentifiable in the frequentist setting e.g. the comparison of c2 vs c3. However, since there is no information on the network for these comparisons, the corresponding posterior estimates will be very uncertain, reflecting the priors used. Thus, having “non-identifiable” parameters in the model does not affect the estimation of identifiable quantities. Moreover, unidentifiable parameters or unidentifiable combinations thereof will be easy to spot by just looking at the posteriors. In the running example, our posteriors will be very uncertain about $d_{2}$ or about $d_{2}-d_{3}$, indicating that we actually know nothing about these relative effects, but will bear evidence about $d_{1}+d_{2}-d_{3}-d_{4}$.

Here is a program in R to showcase all this, using the example above:

# GENERATE DATA EXAMPLE 1---------------------------------------------------------------

# the first study compares c1+c2 vs c2

# the second study compares c3+c4 vs c4

# the third study compares c1+c2 vs c3+c4

dat1=data.frame(study=c(1,2,3), c1=c(1,0,1), c2=c(0,0,1),

c3=c(0,1,-1), c4=c(0,0,-1),y=c(1,1,0), se=c(1,1,1))

# NO INTERACTION CNMA MODEL ----------------------------------------------------------

model1.string <- "

model {

for(i in 1:Ns) {

prec[i]<-1/(se[i]*se[i])

y[i]~dnorm(mean[i],prec[i])

mean[i]<- inprod(d[], c[i,])

}

##prior distributions

for(i in 1:Nc){d[i]~dnorm(0,0.001)}

#examples

example1<- d[1]+d[2]-d[3]-d[4]

example2<- d[2]-d[3]

}

"

model1.spec<-textConnection(model1.string)

data <- list(y=dat1$y,se=dat1$se, Nc=4, Ns=3, c=dat1[,2:5])

jags.m=0

jags.m <- jags.model(model1.spec, data = data, n.chains =4, n.adapt = 10000)

params <- c("d", "example1" , "example2" )

samps<- coda.samples(jags.m, params, n.iter =10000)

MCMCtrace(samps,pdf = FALSE, params = 'd[1]',ISB = FALSE, exact = TRUE)

MCMCtrace(samps,pdf = FALSE, params = 'd[2]',ISB = FALSE, exact = TRUE)

MCMCtrace(samps,pdf = FALSE, params = 'example1',ISB = FALSE, exact = TRUE)

MCMCtrace(samps,pdf = FALSE, params = 'example2',ISB = FALSE, exact = TRUE)

MCMCsummary(samps)

And here are results:

mean sd 2.5% 50% 97.5% Rhat n.eff

d[1] 1.0000 0.99 -0.94 1.0e+00 2.9 1.0 13459

d[2] -4.9285 23.65 -49.15 -5.1e+00 44.3 1.3 43

d[3] 0.9952 1.00 -0.97 1.0e+00 2.9 1.0 12874

d[4] -4.9219 23.62 -48.97 -5.2e+00 44.1 1.3 43

example1 -0.0017 1.00 -1.96 1.3e-05 2.0 1.0 40292

example2 -5.9237 23.65 -50.05 -6.2e+00 43.2 1.3 43

As expected, d[1] and d[2] are estimated with accuracy, while d[2] and d[4] have very wide posteriors (in fact identical to the priors). Example1 corresponds to the comparison c1+c2 vs. c3+c4 (identifiable from the data), while example2 to the comparison c2 vs. c3 (unidentifiable). Let us also look at some of the posteriors:


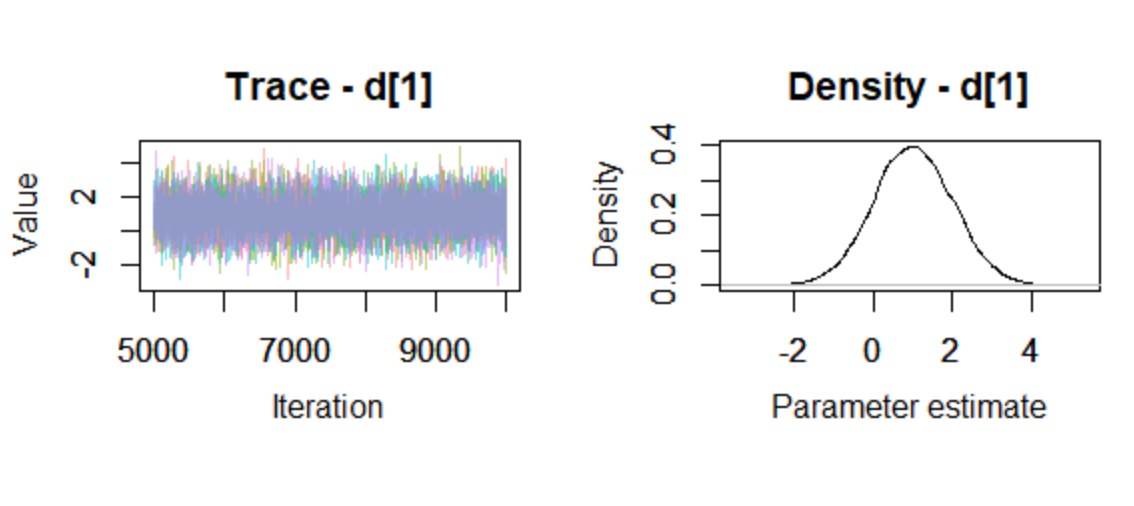


**
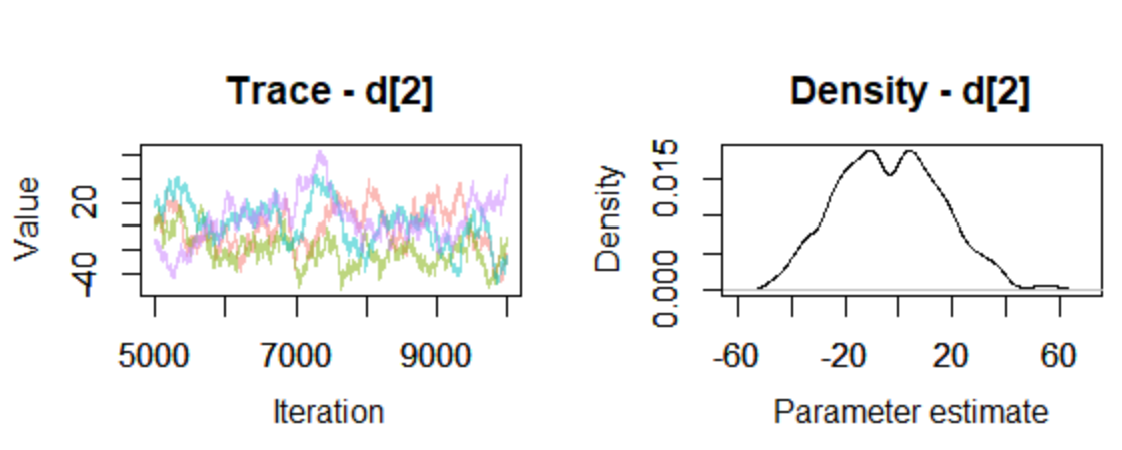
**


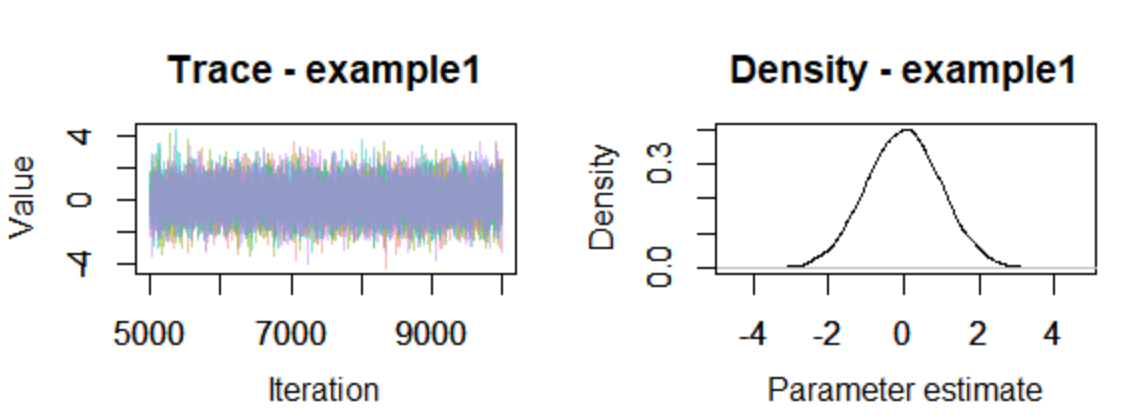


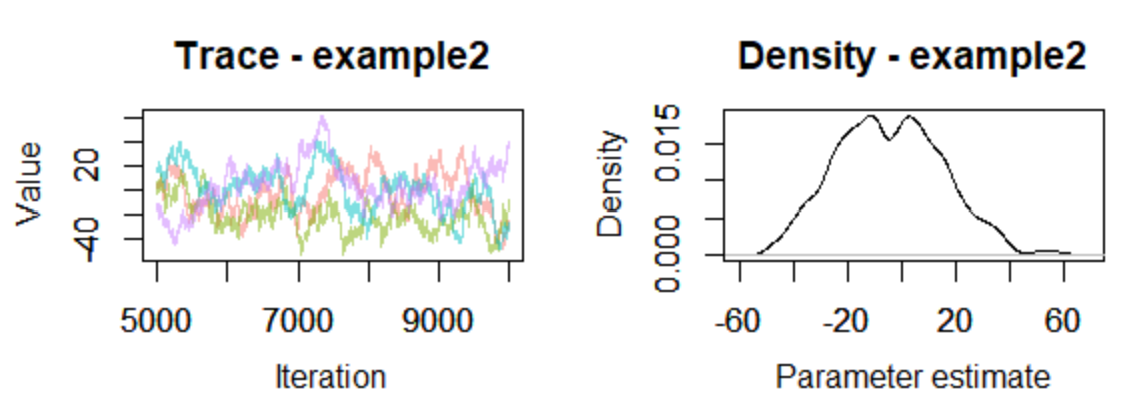


As we can see also from the plots, $d_{1}$ and example1 can be estimated, but the posteriors for $d_{2}$ and example2 are wide, non-smooth and the chains do not converge. Thus, we see that non-identifiable components do not affect the estimation of identifiable ones. Also, we can have non-identifiable components, but identifiable combinations thereof. In practical applications, just looking at the posterior distributions offers a simple way to assess identifiability: quantities that are unidentifiable given the data and the model, will simply have wide and erratic posterior distributions, with no convergence of the independent chains.

## *Component NMA model with interactions*

Let us now move to the case where we want to include interactions among components in the model. Let us assume that we have 3 components and only 4 studies in the network.

- Study 1 compares c1 vs. c2.
- Study 2 compares c1 vs. c3.
- Study 3 compares c1+c2 vs. c3.
- Study 4 compares c1+c2+c3 vs. c3.

If we use an additive model, the effects of all components are identifiable. If we use a model with all two-way interactions, we have in total 6 parameters in the model: $d_{1}$, $d_{2}$, $d_{3}$, $d_{1.2}, d_{1.3}, d_{2.3}$. Obviously, we cannot identify 6 parameters from these 4 trials. However, relative effects between combinations of components (i.e. combinations of these 6 parameters) may still be identifiable. Also, as we will show in this example, some effects may be better estimated using a penalized interactions model as compared to the additive model or the unpenalized interactions model.

To illustrate all these, let us assume that the true values for the parameters are $d_{1}=1$, $d_{2}=2$, $d_{3}=3$, $d_{1.2}=1, d_{1.3}= d_{2.3}=0$ (i.e. only interaction between c1 and c2) and that all studies provide a perfectly accurate estimate of the corresponding effect. Let us use the additive model, the interaction model with vague priors for the interactions, and also an SSVS model to analyse the data.

Let us also try to estimate the following comparisons:

- c1+c2 vs c2. The true value is $d_{1}+d_{1.2}+d_{2}-d_{3}=2$.
- c2+c3 vs c3. The true value is $d_{2}+d_{3}-d_{3}=2$.
- c1 vs c2. The true value is $d_{1}-d_{2}=-1.$
- c1+c2 vs c1+c3. The true value is $d_{1}+d_{2}+d_{1.2}-d_{1}-d_{3}=0$.
- c1+c2 vs c2+c3. The true value is $d_{1}+d_{2}+d_{1.2}-d_{2}-d_{3}=-1$.

Example code in R below:

# GENERATE DATA EXAMPLE 2------------------------------------------------------

# four studies available

# the first compares c1 vs c2

# the second compares c1 vs c3

# the third compares c1 + c2 vs c3

# the fourth compares c1 + c2 + c3 vs c3

dat2=data.frame(study=c(1,2,3, 4), c1=c(1,1,1,1), c2=c(-1,0,1,1),

c3=c(0,-1,-1,0), c12=c(0,0,1,1),c13=c(0,0,0,1), c23=c(0,0,0,1),

y=c(-1,-2,1,4), se=c(0.1,0.1,0.1,0.1))

# NO INTERACTION MODEL ---------------------------------------------------------

model1.string <- "

model {

for(i in 1:Ns) {

prec[i]<-1/(se[i]*se[i])

y[i]~dnorm(mean[i],prec[i])

mean[i]<- inprod(d[], c[i,])}

##prior distributions

for(i in 1:Nc){d[i]~dnorm(0,0.001)}

#examples

example1<- d[1] ### compare c1+c2 vs c2

example2<- d[2] ### compare c2+c3 vs c3

example3<- d[1]-d[2] ### compare c1 vs c2

example4<- d[2]-d[3] ### compare c1+c2 vs c1+c3

example5<- d[1]-d[3] ### compare c1+c2 vs c2+c3

}

"

model1.spec<-textConnection(model1.string)

data <- list(y=dat2$y,se=dat2$se, Nc=3, Ns=4, c=dat2[,2:4])

jags.m=0

jags.m <- jags.model(model1.spec, data = data, n.chains =3, n.adapt = 5000)

params <- c("d", "example1" , "example2", "example3", "example4", "example5")

samps<- coda.samples(jags.m, params, n.iter =5000)

MCMCtrace(samps,pdf = FALSE, params = 'd[1]',ISB = FALSE, exact = TRUE)

MCMCtrace(samps,pdf = FALSE, params = 'd[2]',ISB = FALSE, exact = TRUE)

MCMCtrace(samps,pdf = FALSE, params = 'example1',ISB = FALSE, exact = TRUE)

MCMCtrace(samps,pdf = FALSE, params = 'example2',ISB = FALSE, exact = TRUE)

m1=MCMCsummary(samps)

m1

# INTERACTION MODEL VAGUE PRIOR -------------------------------------------------

model2.string <- "

model {

for(i in 1:Ns) {

prec[i]<-1/(se[i]*se[i])

y[i]~dnorm(mean[i],prec[i])

mean[i]<- inprod(d[], c[i,])+d12*c12[i]+d13*c13[i]+d23*c23[i]}

##prior distributions

for(i in 1:Nc){d[i]~dnorm(0,0.001)}

d12~dnorm(0,0.001)

d13~dnorm(0,0.001)

d23~dnorm(0,0.001)

#examples

example1<- d[1]+d12 ### compare c1+c2 vs c2

example2<- d[2]+d23 ### compare c2+c3 vs c3

example3<- d[1]-d[2] ### compare c1 vs c2

example4<- d[2]+d12-d[3] ### compare c1+c2 vs c1+c3

example5<- d[1]+d12-d[3] ### compare c1+c2 vs c2+c3}

"

model2.spec<-textConnection(model2.string)

data <- list(y=dat2$y,se=dat2$se, Nc=3, Ns=4, c=dat2[,2:4], c12=dat2$c12, c13=dat2$c13, c23=dat2$c23)

jags.m=0

jags.m <- jags.model(model2.spec, data = data, n.chains =3, n.adapt = 5000)

params <- c("d","d12","d23","d13","example1" , "example2", "example3", "example4", "example5")

samps<- coda.samples(jags.m, params, n.iter =5000)

MCMCtrace(samps,pdf = FALSE, params = 'd[1]',ISB = FALSE, exact = TRUE)

MCMCtrace(samps,pdf = FALSE, params = 'd[3]',ISB = FALSE, exact = TRUE)

MCMCtrace(samps,pdf = FALSE, params = 'example1',ISB = FALSE, exact = TRUE)

MCMCtrace(samps,pdf = FALSE, params = 'd12',ISB = FALSE, exact = TRUE)

m2=MCMCsummary(samps)

# INTERACTION MODEL SSVS --------------------------------------------------------

model3.string <- "

model {

for(i in 1:Ns) {

prec[i]<-1/(se[i]*se[i])

y[i]~dnorm(mean[i],prec[i])

mean[i]<- inprod(d[], c[i,])+d12*c12[i]+d13*c13[i]+d23*c23[i]}

##prior distributions

for(i in 1:Nc){d[i]~dnorm(0,0.001)}

## SSVS

for(k in 1:3){

IndA[k] ~ dcat(Pind[])

Ind[k] <- IndA[k] - 1}

d12 ~ dnorm(0, tauCov[IndA[1]])

d13 ~ dnorm(0, tauCov[IndA[2]])

d23 ~ dnorm(0, tauCov[IndA[3]])

zeta <- pow(eta, -2)

eta ~ dnorm(0,1000)I(0,)

tauCov[1] <- zeta

tauCov[2] <- zeta * 0.01 # g = 100

Pind[1] <- 0.5

Pind[2] <- 0.5

#examples

example1<- d[1]+d12 ### compare c1+c2 vs c2

example2<- d[2]+d23 ### compare c2+c3 vs c3

example3<- d[1]-d[2] ### compare c1 vs c2

example4<- d[2]+d12-d[3] ### compare c1+c2 vs c1+c3

example5<- d[1]+d12-d[3] ### compare c1+c2 vs c2+c3

}

"

model3.spec<-textConnection(model3.string)

data <- list(y=dat2$y,se=dat2$se, Nc=3, Ns=4, c=dat2[,2:4], c12=dat2$c12, c13=dat2$c13, c23=dat2$c23)

jags.m=0

jags.m <- jags.model(model3.spec, data = data, n.chains =3, n.adapt = 5000)

params <- c("d","d12","d23","d13","example1" , "example2", "example3", "example4", "example5")

samps<- coda.samples(jags.m, params, n.iter =5000)

MCMCtrace(samps,pdf = FALSE, params = 'd[1]',ISB = FALSE, exact = TRUE)

MCMCtrace(samps,pdf = FALSE, params = 'd[3]',ISB = FALSE, exact = TRUE)

MCMCtrace(samps,pdf = FALSE, params = 'example1',ISB = FALSE, exact = TRUE)

MCMCtrace(samps,pdf = FALSE, params = 'd12',ISB = FALSE, exact = TRUE)

m3=MCMCsummary(samps)

m1

m2

m3

Results for the additive model are as follows:

mean sd 2.5% 50% 97.5% Rhat n.eff

d[1] 1.5 0.073 1.36 1.5 1.64 1 9526

d[2] 2.6 0.064 2.47 2.6 2.72 1 21678

d[3] 3.3 0.111 3.09 3.3 3.51 1 8932

example1 1.5 0.073 1.36 1.5 1.64 1 9526

example2 2.6 0.064 2.47 2.6 2.72 1 21678

example3 -1.1 0.096 -1.29 -1.1 -0.91 1 15706

example4 -0.7 0.110 -0.91 -0.7 -0.49 1 11306

example5 -1.8 0.080 -1.95 -1.8 -1.65 1 19112

Results for the interaction model with flat priors:

mean sd 2.5% 50% 97.5% Rhat n.eff

d[1] 1.959 3.96 -6.6 2.87 8.0 7.1 7

d[2] 2.959 3.96 -5.6 3.86 9.0 7.1 7

d[3] 3.959 3.96 -4.7 4.88 10.0 7.1 8

d12 0.041 3.96 -5.9 -0.84 8.7 7.0 8

d13 -9.027 5.98 -21.2 -9.00 3.3 2.1 9

d23 8.068 6.85 -1.7 6.53 21.8 3.7 11

example1 2.000 0.17 1.7 2.00 2.3 1.0 2618

example2 11.028 5.98 -1.3 10.99 23.2 2.1 8

example3 -1.000 0.10 -1.2 -1.00 -0.8 1.0 9016

example4 -0.959 3.96 -6.9 -1.85 7.7 7.0 7

example5 -1.959 3.96 -7.9 -2.85 6.7 7.0 7

Results for the interaction model with SSVS are as follows:

mean sd 2.5% 50% 97.5% Rhat n.eff

d[1] 1.58 0.35 0.86 1.614 2.17 1 181

d[2] 2.61 0.35 1.85 2.663 3.17 1 179

d[3] 3.52 0.37 2.81 3.523 4.21 1 255

d12 0.26 0.37 -0.19 0.116 1.17 1 250

d13 -0.21 0.33 -0.99 -0.074 0.25 1 640

d23 -0.22 0.34 -1.01 -0.071 0.24 1 559

example1 1.84 0.18 1.49 1.846 2.21 1 1315

example2 2.40 0.33 1.76 2.392 3.03 1 600

example3 -1.03 0.10 -1.23 -1.030 -0.83 1 5579

example4 -0.64 0.36 -1.20 -0.712 0.17 1 195

example5 -1.67 0.36 -2.18 -1.768 -0.84 1 203

By comparing the true values of the 5 examples (2,2,-1,0,1) with the estimated ones, we see that although there were not enough data to estimate all parameters, the SSVS provided less biased estimations for all relative effects, as compared to the additive model, or compared to the flat prior interaction model. The latter completely failed to estimate the relative effects of example2, example4 and example5.

# **Possible issues related to stepwise methods for selecting interactions in component network meta-analysis**

Rücker et al.^2^ recently described a method for deciding on whether the additive model or models with interactions provide better fit to the data, as compared to the standard NMA model. Rücker et al^3^. also described two stepwise model selection methods (a forward and a backward method) for deciding which interaction(s) to include in the model. These methods perform tests using Cochran’s Q statistic to compare different models.

A practical problem that may come up with this approach is that the number of tests that need to be performed can be large for networks with many components comparing many different combinations For example, if we assume a big network with treatments including a total of 15 components, there are $15\times14/2=105$ different models that include a single two-way interaction term between the components. There are $105\times104/2=5460$ models including only two two-way interaction terms that we can fit. For more components this number obviously blows up, while if we include covariate-component interactions things become even more unmanageable. Of course, in practical applications, most interactions between components in a network will be unidentifiable due to data limitations; even so, there may still be a large number of models we can choose from.

Moreover, it has been suggested that the use of stepwise variable selection methods in regression modelling may lead to suboptimal model performance in some cases; for a summary of potential issues associated with stepwise variable selection methods we refer to Section 4.3 in the book by Harrell^4^, and Section 11.4 in the book by Steyerberg^5^. To clarify this issue, let us assume that interaction effects are generally small (i.e. considerably smaller than the main effects of components), and that there is some variability of these effects across trials. A stepwise variable procedure will tend to include in the model interaction terms that happened to be large in the particular sample at hand, and will remove terms that happened to be small. This will lead to an overestimation of the effects of the selected interaction terms (“winner’s curse”, also related to regression to the mean). Moreover, even though some interactions may be small when seen in isolation (and thus removed in a stepwise approach), their combined effect may be large. Another general issue with stepwise methods is that they disregard the uncertainty of the model selection procedure. In other words, the standard errors of all model’s parameters in a stepwise procedure are obtained as if the final model was prespecified, disregarding all intermediate steps. This will generally lead to underestimation of all standard errors, and to overconfident results^6^.

On the other hand, including all interaction terms in the model, e.g. by assigning vague prior distributions to all $d_{p.q}$ of model IV, will lead to estimates of relative effects with very large credible intervals, since for many interactions there will be only limited information on the data.

# **Utilizing an IPD-NMA model in clinical practice**

An IPD-NMA model (such as model II) can be used to estimate relative treatment effects for every treatment comparison and for any combination of patient covariates. Furthermore, IPD-CNMA models (such as models VII and VIII) have the capacity to do so for any combination of components. Thus, such models could be used to inform the choice of treatments in clinical practice at the patient level; this might be particularly important in some medical fields, where assigning a patient to an intervention is often a matter of trial and error. A doctor could use such a model to input $\boldsymbol{x}$ (patient covariates) and estimate all relative effects among all available treatments, or among all possible combinations of components.

However, using such a Bayesian model poses practical problems: fitting an IPD-CNMA model for the depression case study (see main paper) took around 30 hours in a laptop computer; moreover, the IPD required to fit such models are usually protected by confidentiality agreements. Thus, it would not be practical to fit such a model real-time, to estimate personalized treatment effects. In most cases it would be also infeasible to provide tables of estimated relative effects among all treatments and for all combinations of covariates for the clinical doctors to use, due to the number of possible combinations of covariates and the number of treatment comparisons.

One way to overcome this problem is, after fitting the model, to make publicly available all MCMC posterior samples of all model’s parameters. These samples can then be used to estimate relative effects for any value of $\boldsymbol{x}$ and all treatment comparisons. An alternative method is as follows: assuming that the posterior estimates of such a model are approximately normal, we can extract from the posterior distribution the means and the variance-covariance matrix for all model’s parameters. Then, we can use these quantities to estimate all treatment effects given $\boldsymbol{x}$. Both methods described in this paragraph could be used, for example, to develop an online web-application where doctors would input patients’ characteristics and obtain relative effect estimates.

An example of the code required to develop such a package can be found in <https://github.com/esm-ispm-unibe-ch/Bayesian-CNMA/blob/master/shiny%20app%20for%20depression.R>. This uses the **shiny** package in R.^7^

# **Results from the IPD CNMA of the depression dataset**

|  | **Depression severity (iMD in PHQ-9 scores)** | | |
| --- | --- | --- | --- |
|  | **Median** | **95% CrI** | |
| **age** | 0.191 | -0.089 | 0.473 |
| **baseline depression** | 2.591 | 2.322 | 2.853 |
| **gender** | -0.034 | -0.281 | 0.176 |
| **relationship** | -0.122 | -0.33 | 0.119 |
| ***wl*** | 0.415 | -0.75 | 1.534 |
| ***dt*** | -0.238 | -61.894 | 61.998 |
| ***pl*** | -1.407 | -2.515 | -0.295 |
| ***pe*** | 0.016 | -0.863 | 0.929 |
| ***cr*** | 0.296 | -0.871 | 1.406 |
| ***ba*** | -1.832 | -2.902 | -0.8 |
| ***is*** | -0.543 | -1.587 | 0.517 |
| ***ps*** | -0.641 | -1.412 | 0.092 |
| ***re*** | 1.202 | 0.168 | 2.274 |
| ***w3*** | -0.528 | -1.552 | 0.492 |
| ***bi*** | -1.817 | -3.922 | 0.259 |
| ***rp*** | 0.349 | -0.685 | 1.323 |
| ***hw*** | 0.309 | -0.689 | 1.347 |
| ***ftf*** | 0.853 | -1.797 | 3.407 |
| ***ae*** | -0.256 | -1.131 | 0.603 |
| ***he*** | -0.292 | -1.173 | 0.583 |
| ***tg*** | 0.007 | -0.88 | 0.889 |
| **age*wl** | 0.019 | -0.14 | 0.31 |
| **age*dt** | 0 | -0.2 | 0.207 |
| **age*pl** | -0.01 | -0.196 | 0.136 |
| **age*pe** | 0 | -0.163 | 0.179 |
| **age*cr** | -0.006 | -0.184 | 0.152 |
| **age*ba** | -0.015 | -0.205 | 0.122 |
| **age*is** | -0.007 | -0.209 | 0.173 |
| **age*ps** | -0.009 | -0.175 | 0.129 |
| **age*re** | -0.011 | -0.192 | 0.137 |
| **age*w3** | -0.013 | -0.228 | 0.14 |
| **age*bi** | -0.009 | -0.234 | 0.153 |
| **age*rp** | -0.007 | -0.17 | 0.129 |
| **age*hw** | 0.007 | -0.151 | 0.249 |
| **age*ftf** | -0.039 | -0.473 | 0.122 |
| **age*ae** | 0.001 | -0.16 | 0.18 |
| **age*he** | -0.023 | -0.328 | 0.135 |
| **age*tg** | -0.014 | -0.277 | 0.22 |
| **baseline depression*wl** | 0.052 | -0.075 | 0.31 |
| **baseline depression*dt** | 0.001 | -0.213 | 0.209 |
| **baseline depression*pl** | 0.01 | -0.136 | 0.241 |
| **baseline depression*pe** | -0.065 | -0.339 | 0.068 |
| **baseline depression*cr** | -0.027 | -0.24 | 0.111 |
| **baseline depression*ba** | -0.063 | -0.338 | 0.072 |
| **baseline depression*is** | -0.046 | -0.335 | 0.079 |
| **baseline depression*ps** | 0.022 | -0.095 | 0.228 |
| **baseline depression*re** | 0.014 | -0.117 | 0.219 |
| **baseline depression*w3** | 0 | -0.18 | 0.185 |
| **baseline depression*bi** | 0 | -0.193 | 0.198 |
| **baseline depression*rp** | -0.004 | -0.157 | 0.147 |
| **baseline depression*hw** | -0.053 | -0.287 | 0.072 |
| **baseline depression*ftf** | 0.091 | -0.039 | 0.347 |
| **baseline depression*ae** | -0.032 | -0.243 | 0.092 |
| **baseline depression*he** | -0.076 | -0.351 | 0.054 |
| **baseline depression*tg** | -0.03 | -0.243 | 0.098 |
| **gender*wl** | -0.024 | -0.231 | 0.099 |
| **gender*dt** | 0 | -0.206 | 0.212 |
| **gender*pl** | 0.028 | -0.097 | 0.258 |
| **gender*pe** | 0.001 | -0.155 | 0.152 |
| **gender*cr** | -0.005 | -0.164 | 0.134 |
| **gender*ba** | 0.002 | -0.158 | 0.151 |
| **gender*is** | -0.014 | -0.215 | 0.121 |
| **gender*ps** | 0.005 | -0.128 | 0.153 |
| **gender*re** | 0.006 | -0.129 | 0.179 |
| **gender*w3** | -0.023 | -0.265 | 0.113 |
| **gender*bi** | 0 | -0.206 | 0.185 |
| **gender*rp** | 0.018 | -0.107 | 0.183 |
| **gender*hw** | 0.054 | -0.064 | 0.293 |
| **gender*ftf** | 0.01 | -0.102 | 0.17 |
| **gender*ae** | 0.002 | -0.152 | 0.149 |
| **gender*he** | 0.016 | -0.113 | 0.188 |
| **gender*tg** | 0.034 | -0.082 | 0.235 |
| **relationship*wl** | 0.001 | -0.15 | 0.153 |
| **relationship*dt** | -0.001 | -0.206 | 0.21 |
| **relationship*pl** | -0.015 | -0.217 | 0.117 |
| **relationship*pe** | -0.009 | -0.178 | 0.126 |
| **relationship*cr** | -0.008 | -0.17 | 0.121 |
| **relationship*ba** | -0.018 | -0.205 | 0.104 |
| **relationship*is** | 0.039 | -0.084 | 0.306 |
| **relationship*ps** | -0.022 | -0.202 | 0.092 |
| **relationship*re** | 0.02 | -0.111 | 0.221 |
| **relationship*w3** | 0.012 | -0.135 | 0.215 |
| **relationship*bi** | -0.008 | -0.248 | 0.158 |
| **relationship*rp** | -0.004 | -0.151 | 0.132 |
| **relationship*hw** | -0.007 | -0.165 | 0.12 |
| **relationship*ftf** | -0.004 | -0.148 | 0.124 |
| **relationship*ae** | 0.01 | -0.115 | 0.177 |
| **relationship*he** | -0.02 | -0.198 | 0.103 |
| **relationship*tg** | -0.003 | -0.156 | 0.138 |

The star symbol * denotes interaction terms. CrI: credible interval.

Common heterogeneity $\tau$ was estimated to be 1.20 (95%CrI: 0.89 to 1.57) in PHQ-9 points.

# **Additional example in multiple myeloma**

## *Description of the dataset*

This dataset includes study-level (aggregate) information from 25 two-armed randomized controlled trials (RCTs), on patients with multiple myeloma. Multiple myeloma constitutes the second most frequent form of blood cancer. At the treatment level, the dataset creates two disconnected networks. However, the treatments in these networks share the same components, so that a component NMA can be performed using information from all studies. More specifically, the treatments were combinations of up to 3 different components, out of a total of 18 components. The components were: bev (bevacizumab), bor (bortezomib), carf (carfilzomib), cyc (cyclophosphamide), dara (daratumumab), dex (dexamethasone), elo (elozumatab), IFN (interferon alpha), ixa (ixazomib), len (lenalidomide), ob (oblimersen), pan (panobinostat), peri (perifosine), PLD (pegylated liposomal doxorubicin), pom (pomalidomide), sil (silituximab), thal (thalidomide), vor (vorinostat). The outcome we focus on in this paper is progression-free survival, measured in the hazard ratio (HR) scale. The dataset was used by Schmitz et al.^8^, and was re-analysed by Rücker et al.^3^ We provide the data in <https://github.com/esm-ispm-unibe-ch/Bayesian-CNMA>. The network at the treatment level is disconnected, and comprises two separate networks. The two networks are shown below, Figure 5. Numbers on edges indicate number of studies performing the corresponding comparison.


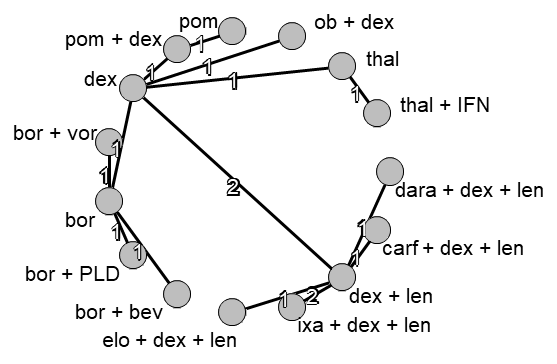

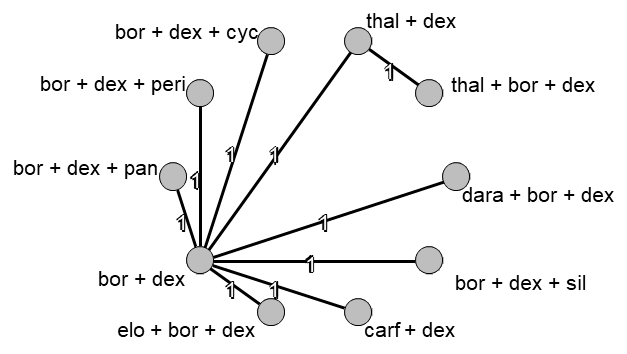


**Figure 5**: Network graph for studies in multiple myeloma

## *Results from the analysis*

In this dataset there were only AD studies providing log hazard ratios (logHR) and corresponding standard errors. We employed three different models for the analysis: (i) model III, the Bayesian additive AD-CNMA model with no interactions; (ii) model V, the AD-CNMA model with SSVS for two-way interactions, where we assumed all interactions to be equiprobable, i.e. $I_{p.q}\sim Bernoulli(0.5)$ for all pairs, and $\eta\sim N\left( 0,\sigma^{2}=0.001 \right), g=100$; and (iii) model VI, the AD-CNMA model with Bayesian LASSO for interactions, where we assumed $\lambda^{-1}\sim U\left( 0,5 \right)$ as a prior distribution. For all analyses we used a vague prior distribution for heterogeneity, i.e. we used the positive part of $N\left( 0, \sigma^{2}=10 \right)$. After fitting each model, we estimated $d_{q}$, i.e. the main effects of components in logHR scale, the standard deviation of heterogeneity ($\tau$), component interactions, and indicator variables (for SSVS only). For illustration purposes, we also estimated the relative effects (in hazard ratios) for the following four treatment comparisons: thal + dex VS thal + bor + dex; bor + dex + peri VS thal + IFN; ob + dex VS dara + dex + len; bor + dex + cyc VS thal + IFN. For the analyses we used four independent chains with 30,000 iterations after 10,000 iterations burn-in.

In Table 1 we provide the estimated values of the main effects $d_{p}$for each component, heterogeneity $\tau$, and the estimated hazard ratios for the four treatment comparisons presented above. We first note that the two variable selection methods gave very similar results, and that the inclusion of interaction terms reduced heterogeneity in the network significantly, as compared to the additive model. Regarding the four treatment comparisons, the inclusion of interaction terms did not affect the point estimate much but led to changes on the width of the credible intervals. Figure 6 shows a scatterplot of the posterior mean of the indicator variables versus the estimated interaction terms, for the SSVS model. The points follow a V-shaped distribution, as expected. Three interactions stand out as potentially important: len-carf, dex-bor and dex-pom. These were the interactions that the SSVS model identified as more important, included in the model 64%, 61% and 58% of the times respectively. These interactions were also picked up by the Bayesian LASSO model, i.e. their estimated coefficients had the largest posterior values. Interactions dex-bor and dex-pom were also identified as important in the frequentist analysis by Rücker et al.^3^ A sensitivity analysis using $\eta\sim N\left( 0,\sigma^{2}=0.0001 \right), g=1000$ for SSVS gave broadly similar results. Likewise, changing the prior for the $\lambda$ parameter of LASSO to $N({0,10}^{2})I(0,)$ had a relatively small impact on the results. Overall, we conclude that the interaction CNMA models with SSVS and Bayesian LASSO gave very similar results. In both cases, the inclusion of interactions between components explained some of the observed heterogeneity, but results did not alter the conclusions drawn regarding the four example comparisons as compared to results obtained from the simpler additive model.

**Table 1:** Estimated log hazard ratios [95% Credible Intervals] for each component (dex, bor, …), heterogeneity standard deviation (τ), and hazard ratios for four treatment comparisons, based on different CNMA models. Abbreviations: component network meta-analysis (CNMA), stochastic search variable selection (SSVS). Abbreviations of components given in text.

| **Estimated quantity** | **Additive CNMA**  **(no interactions)** | **CNMA with SSVS** | **CNMA with Bayesian LASSO** |
| --- | --- | --- | --- |
| dex | -0.06 [-0.66; 0.47] | -0.08 [-0.69; 0.53] | -0.08 [-0.85; 0.74] |
| bor | -0.27 [-0.80; 0.21] | -0.41 [-1.14; 0.18] | -0.49 [-1.36; 0.28] |
| thal | -0.37 [-1.12; 0.31] | -0.36 [-1.06; 0.34] | -0.33 [-1.16; 0.55] |
| len | -0.86 [-1.37; -0.36] | -0.86 [-1.55; -0.20] | -0.85 [-2.00; 0.49] |
| pom | -0.75 [-1.45; -0.05] | -0.66 [-1.36; 0.18] | -0.59 [-1.40; 0.46] |
| PLD | -0.33 [-1.01; 0.36] | -0.32 [-1.19; 0.46] | -0.32 [-1.56; 0.92] |
| bev | -0.19 [-1.04; 0.65] | -0.19 [-1.17; 0.74] | -0.20 [-1.45; 1.03] |
| vor | -0.11 [-0.80; 0.58] | -0.10 [-0.91; 0.65] | -0.11 [-1.23; 1.22] |
| IFN | 1.65 [ 0.40; 2.89] | 1.64 [ 0.32; 2.92] | 1.64 [ 0.01; 3.19] |
| carf | -0.69 [-1.24; -0.15] | -0.75 [-1.58; -0.02] | -0.77 [-2.63; 0.35] |
| ob | 0.14 [-0.61; 0.89] | 0.14 [-0.70; 0.97] | 0.13 [-1.29; 1.26] |
| elo | -0.28 [-0.80; 0.24] | -0.28 [-1.08; 0.39] | -0.29 [-1.60; 0.96] |
| ixa | -0.39 [-0.92; 0.13] | -0.38 [-1.24; 0.43] | -0.38 [-1.90; 1.25] |
| dara | -1.01 [-1.49; -0.52] | -1.00 [-1.72; -0.24] | -0.99 [-2.03; 0.35] |
| sil | -0.07 [-0.79; 0.66] | -0.06 [-0.97; 0.93] | -0.06 [-1.70; 1.63] |
| peri | 0.48 [-0.32; 1.28] | 0.48 [-0.59; 1.48] | 0.49 [-1.19; 2.19] |
| pan | -0.40 [-1.08; 0.28] | -0.39 [-1.27; 0.50] | -0.39 [-1.97; 1.23] |
| cyc | 0.24 [-0.64; 1.11] | 0.23 [-0.82; 1.33] | 0.25 [-1.38; 1.91] |
| τ (heterogeneity standard deviation) | 0.27 [0.13; 0.62] | 0.20 [0.01; 0.55] | 0.15 [0.01; 0.57] |
| **Estimated hazard ratios for example comparisons** | | | |
| (thal + dex) VS (thal + bor + dex) | 1.31 [0.81; 2.22] | 1.31 [0.83; 2.11] | 1.32 [0.81; 2.16] |
| (bor + dex + peri) VS (thal + IFN) | 0.32 [0.06; 1.65] | 0.32 [0.07; 1.50] | 0.32 [0.07; 1.59] |
| (ob + dex) VS (dara + dex + len) | 7.46 [2.68; 20.81] | 7.59 [3.09; 18.27] | 7.66 [3.20; 18.15] |
| (bor + dex + cyc) VS (thal + IFN) | 0.25 [0.05; 1.34] | 0.25 [0.05; 1.26] | 0.26 [0.05; 1.28] |


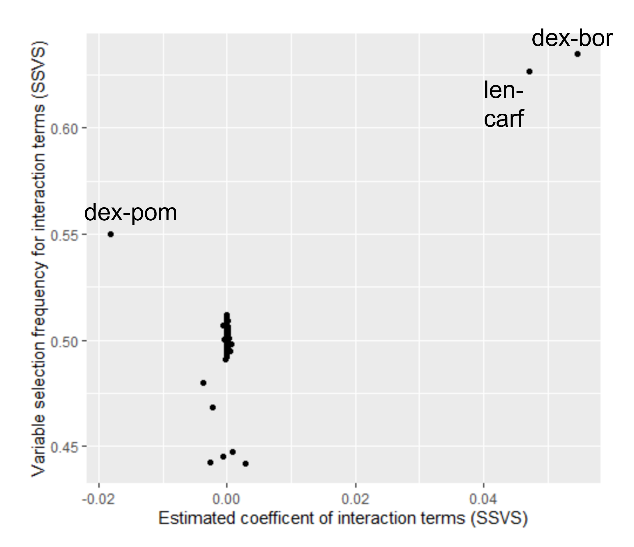


**Figure 6**: Results for component interactions from the Stochastic Search Variable Selection (SSVS) model for the multiple myeloma example. x-axis: estimated interaction terms (log hazard ratios); y-axis: corresponding frequency of selection for the interaction term (i.e. percent of times the interaction term was included in the model). The three most prominent interactions are labelled. Component abbreviations given in text.

# **References**

1. Pompoli A, Furukawa TA, Efthimiou O, Imai H, Tajika A, Salanti G. Dismantling cognitive-behaviour therapy for panic disorder: a systematic review and component network meta-analysis. *Psychological Medicine*. 2018;48(12):1945-1953. doi:10.1017/S0033291717003919

2. Rücker G, Petropoulou M, Schwarzer G. Network meta-analysis of multicomponent interventions. *Biom J*. 2020;62(3):808-821. doi:10.1002/bimj.201800167

3. Rücker G, Schmitz S, Schwarzer G. Component network meta-analysis compared to a matching method in a disconnected network: A case study. *Biometrical Journal*. n/a(n/a). doi:10.1002/bimj.201900339

4. Harrell F. *Regression Modeling Strategies: With Applications to Linear Models, Logistic and Ordinal Regression, and Survival Analysis*. 2nd ed. Springer International Publishing; 2015. doi:10.1007/978-3-319-19425-7

5. Steyerberg E. *Clinical Prediction Models: A Practical Approach to Development, Validation, and Updating*. Springer-Verlag; 2009. doi:10.1007/978-0-387-77244-8

6. Smith G. Step away from stepwise. *J Big Data*. 2018;5(1):32. doi:10.1186/s40537-018-0143-6

7. Chang W, Cheng J, Allaire JJ, et al. *Shiny: Web Application Framework for R*.; 2021. Accessed December 9, 2021. https://CRAN.R-project.org/package=shiny

8. Schmitz S, Maguire Á, Morris J, et al. The use of single armed observational data to closing the gap in otherwise disconnected evidence networks: a network meta-analysis in multiple myeloma. *BMC Med Res Methodol*. 2018;18(1):66. doi:10.1186/s12874-018-0509-7
